# PIM Kinases as Potential Therapeutic Targets in a Subset of Peripheral T Cell Lymphoma Cases

**DOI:** 10.1371/journal.pone.0112148

**Published:** 2014-11-11

**Authors:** Esperanza Martín-Sánchez, Lina Odqvist, Socorro M. Rodríguez-Pinilla, Margarita Sánchez-Beato, Giovanna Roncador, Beatriz Domínguez-González, Carmen Blanco-Aparicio, Ana M. García Collazo, Esther González Cantalapiedra, Joaquín Pastor Fernández, Soraya Curiel del Olmo, Helena Pisonero, Rebeca Madureira, Carmen Almaraz, Manuela Mollejo, F. Javier Alves, Javier Menárguez, Fernando González-Palacios, José Luis Rodríguez-Peralto, Pablo L. Ortiz-Romero, Francisco X. Real, Juan F. García, James R. Bischoff, Miguel A. Piris

**Affiliations:** 1 Molecular Pathology Programme, Spanish National Cancer Research Centre (CNIO), Madrid, Spain; 2 Cancer Genomics Group, Marqués de Valdecilla Research Institute (IDIVAL) & Pathology Department, Hospital Universitario Marqués de Valdecilla, Santander, Spain; 3 Pathology Department, Fundación Jiménez Díaz, Madrid, Spain; 4 Onco-hematology Area, Instituto de Investigación Sanitaria Hospital Universitario Puerta de Hierro - Majadahonda, Madrid, Spain; 5 Monoclonal Antibodies Core Unit, Spanish National Cancer Research Centre (CNIO), Madrid, Spain; 6 Experimental Therapeutics Programme, Spanish National Cancer Research Centre (CNIO), Madrid, Spain; 7 Pathology Department, Hospital Virgen de la Salud, Toledo, Spain; 8 Pathology Department, Hospital La Paz, Madrid, Spain; 9 Pathology Department, Hospital Gregorio Marañón, Madrid, Spain; 10 Pathology Department, Hospital Ramón y Cajal, Madrid, Spain; 11 Pathology Department, 12 de Octubre University Hospital, Medical School Universidad Complutense, Instituto i+12, Madrid, Spain; 12 Dermatology Department, 12 de Octubre University Hospital, Medical School Universidad Complutense, Instituto i+12, Madrid, Spain; 13 Translational Research Laboratory, M. D. Anderson Cancer Center Madrid, Madrid, Spain; University of Navarra, Center for Applied Medical Research , Spain

## Abstract

Currently, there is no efficient therapy for patients with peripheral T cell lymphoma (PTCL). The Proviral Integration site of Moloney murine leukemia virus (PIM) kinases are important mediators of cell survival. We aimed to determine the therapeutic value of PIM kinases because they are overexpressed in PTCL patients, T cell lines and primary tumoral T cells. PIM kinases were inhibited genetically (using small interfering and short hairpin RNAs) and pharmacologically (mainly with the pan-PIM inhibitor (PIMi) ETP-39010) in a panel of 8 PTCL cell lines. Effects on cell viability, apoptosis, cell cycle, key proteins and gene expression were evaluated. Individual inhibition of each of the PIM genes did not affect PTCL cell survival, partially because of a compensatory mechanism among the three PIM genes. In contrast, pharmacological inhibition of all PIM kinases strongly induced apoptosis in all PTCL cell lines, without cell cycle arrest, in part through the induction of DNA damage. Therefore, pan-PIMi synergized with Cisplatin. Importantly, pharmacological inhibition of PIM reduced primary tumoral T cell viability without affecting normal T cells *ex vivo*. Since anaplastic large cell lymphoma (ALK+ ALCL) cell lines were the most sensitive to the pan-PIMi, we tested the simultaneous inhibition of ALK and PIM kinases and found a strong synergistic effect in ALK+ ALCL cell lines. Our findings suggest that PIM kinase inhibition could be of therapeutic value in a subset of PTCL, especially when combined with ALK inhibitors, and might be clinically beneficial in ALK+ ALCL.

## Introduction

Peripheral T cell lymphomas (PTCL) are a very aggressive and heterogeneous group of hematological malignancies [1], [2]. Very little is known about their molecular biology, and consequently, the search for efficient therapies that would improve the outcome of these patients remains challenging [3]. Several factors are responsible for our limited knowledge, such as the low incidence of PTCL, the heterogeneity of its subtypes and the few representative models (cell lines or mouse models) available. It is worth to note that majority of the available cell lines cover very few PTCL subtypes, and are mostly derived from cutaneous T cell lymphomas, including its two most prevalent forms: Sézary Syndrome and Mycosis Fungoides [4].

Among the different PTCL subtypes, PTCL-Not Otherwise Specified (PTCL-NOS), Angioimmunoblastic T-cell Lymphoma (AITL) and Anaplastic Large Cell Lymphoma (ALCL) are the most frequent ones. Within the ALCL group, there are two subgroups, depending on the presence or absence of the chromosomal translocation t(2; 5) (p23; q35), which involves the *ALK* and *NPM1* genes and leads to the overexpression of the fusion protein NPM-ALK [3]. This is considered to be the main oncogenic force in ALK+ ALCL, because it activates the Jak/STAT pathway [5], [6]. The ALK+ ALCL is the only PTCL subgroup with a relatively good prognosis [7], however, around 40% of ALK+ ALCL patients fail to be cured using standard therapeutic approaches [3]. New drugs, such as the ALK inhibitor Crizotinib, seem to improve the survival in these patients in early clinical trials [8].

Although different histological subtypes of PTCL have been identified, the treatment approach has been essentially based on the application of anthracycline-based combination chemotherapy, resulting in poor outcomes [9]. To date, only 3 agents have been recently approved by the FDA for the treatment of relapsed or refractory PTCL: pralatrexate, romidepsin and brentuximab vedotin [9], [10]. Nevertheless, the development of new, efficiently targeted therapies is of great importance to PTCL patients [9], [11], [12].

The Proviral Integration site of the Moloney murine leukemia virus (PIM) family is an important mediator of cell survival, comprising three ubiquitously expressed serine/threonine kinases (PIM1, PIM2 and PIM3) with a broad range of cellular substrates that promote cell growth, proliferation and drug resistance. They are overexpressed in a number of human cancers and frequently associated with poor prognosis in most hematological malignancies [13]. PIM kinases are typically induced by the activation of transcription factors downstream of growth factors, cytokines and mitogenic stimuli signaling pathways, such as the Jak/STAT and NF-κB [13], [14], and are also protected from proteasomal degradation by HSP-70 and HSP-90 [15]. Their activities are mediated through the phosphorylation of a number of proteins, including regulators of transcription (MYC, MYB, RUNX1, RUNX3), cell cycle (p21, p27, CDC25A, CDC25C), protein translation (EIF4E, 4E-BP1), apoptosis (BAD, BCL2, ASK1), signaling intermediates (SOCS1, SOCS3, MAP3K5, mTOR, AKT), and drug resistance proteins (ABC transporters) [13], [14], [15].

Studies using transgenic mice have shown that PIM kinases cooperated with important genes involved in B- and T-cell lymphomagenesis, such as, c-Myc, BCL6 and E2A–PBX1 [14]. On the other hand, triple *PIM1-PIM2-PIM3* knockout mice have been reported to be viable, fertile, and just smaller compared with wild type littermates [13], [14], [15]. Very recently, an abnormal hematopoiesis has been described in these triple-knockout mice [16]. These findings indicate that PIM kinases are important for lymphomagenesis and their absence is well tolerated, suggesting that selective PIM kinase inhibitors might have a low toxicity profile [13]. Based on this, along with some differences in the structural conformation of the ATP-binding pocket in the active site compared with other kinases, PIM kinases have been proposed as promising therapeutic targets for pharmacological inhibition. So far, a number of small molecule inhibitors have been tested *in vitro*, but clinical data are only available for a handful of them. One of the most promising PIM inhibitors (PIMi) was SGI-1776, a compound with activity against PIM1, PIM2 and PIM3 at nanomolar concentrations [13], [14], [15], which induced apoptosis at micromolar doses in chronic lymphocytic leukemia [17], mantle cell lymphoma [18], and acute myeloid leukemia [19]. Unfortunately, the phase I clinical trial of this compound was discontinued in November 2010 because of a strong cardiotoxic effect that impaired its further development [15], [20].

This study aimed to determine the efficiency of PIM kinase inhibition in PTCL, to explore the molecular response of PTCL cells to pharmacological pan-PIM inhibition and to identify those PTCL subgroups that are more susceptible to PIM inhibition.

## Materials and Methods

### Ethics statement

The research was approved by the Hospital Universitario Marqués de Valdecilla ethics committee (Santander, Spain).

All the human samples used in this study have been procured from the Spanish CNIO Biobank, located in the Spanish National Cancer Research Centre (Madrid, Spain) (https://www.cnio.es/ing/servicios/biobanco/index.asp), and according to the Spanish legal framework regarding written informed consent and sample anonymization.

Additionally, some samples used here were also previously used in [15].

### Bioinformatics analysis in the PTCL patient series

The gene expression profiles of frozen tumoral samples from 38 PTCL patients and 6 reactive lymph nodes were compared using microarrays. Briefly, differentially expressed genes between PTCL and reactive lymph nodes were identified using a t-test. Then, Gene Set Enrichment Analysis (GSEA) ranked them according to its correlation with *PIM1* or *PIM2* expression. More details are provided in the Supplementary Information (Methods S1). All raw microarray data regarding the PTCL patient series are available at the Gene Expression Omnibus under accession number GSE36172.

### Cell lines, primary samples and reagents

Eight human PTCL cell lines were used in this study. HH (cutaneous T cell lymphoma) and MJ (HTLV1+ PTCL) were obtained from the American Type Cell Collection (ATCC, Rockville, MD); MyLa (Mycosis Fungoides) and HuT78 (Sézary Syndrome) were obtained from the European Collection of Cell Cultures (ECACC, Salisbury, UK); DERL7 (hepatosplenic gamma-delta T cell lymphoma) and SR786, KARPAS-299 and SU-DHL-1 (ALK+ ALCL) were obtained from the German Collection of Microorganisms and Cell Cultures (DSMZ, Braunschweig, Germany). All of them except MJ were cultured in RPMI 1640 medium (IMDM medium for MJ cells) supplemented with 10% heat-inactivated fetal bovine serum (FBS) and 1% penicillin/streptomycin (all from Life Technologies, Carlsbad, CA) in a humidified atmosphere at 37°C and 5% CO_2_. The DERL7 cell line was supplemented with 20% FBS and 20 ng/ml human IL-2 (PeproTech, Rocky Hill, NJ). Cell lines were previously authenticated by DSMZ (year 2010–2011).

Human primary samples were used to measure the basal *PIM1, PIM2* and *PIM3* mRNA levels. Tumoral and normal T cells were respectively isolated from the peripheral blood of 5 Sézary Syndrome patients and 3 healthy donors, through negative selection using the RosetteSep kit (StemCell Technologies, Grenoble, France). Sample purity was checked by flow cytometry, and an enrichment of >90% of CD3+ cells was ensured in all samples. Additionally, the PIM inhibitor sensitivity of primary T cells from 8 PTCL patients (5 Sézary Syndrome and 3 Mycosis Fungoides) and 5 healthy donors was tested.

The pan-PIM inhibitors ETP-39010 [21], ETP-47652, ETP-47551 and ETP-46638 were developed by the Experimental Therapeutics Programme of the Spanish National Cancer Research Centre (Madrid, Spain). The chemical structure of these compounds has been published in [22] under publication number WO 2011/080510. (http://www.wipo.int/portal/index.html.en). The ALK inhibitor (ALKi) Crizotinib was obtained from Selleck Chemicals (Houston, TX). All inhibitors were dissolved in DMSO and the stocks were kept at −20°C. They were diluted in culture medium at desired concentrations immediately before use. For the controls DMSO concentration in the medium was lower than 0.2%.

### PIM genetic silencing experiments

Transient genetic silencing was performed in PTCL cell lines as follows: HH, SR786, SU-DHL-1 and MyLa cell lines were electroporated with specific small interference RNAs (siRNAs) against *PIM1*, *PIM2* and *PIM3* genes, using the Neon Transfection System (Life Technologies) and following the manufacturer’s instructions, as previously described [23]. Briefly, cells were incubated without antibiotics overnight and resuspended in R buffer at a density of 500,000 cells/ml. Then, siPIM1 (s10527), siPIM2 (s21749), siPIM3 (HSS140560) and the Non-Template Control (NTC, AM4635) (all from Life Technologies) were added to the cells at several concentrations (25–100 nM). Microporation conditions were set up for each cell line (900 V, 30 ms and 2 pulses for HH, SR786 and SU-DHL-1; 1300 V, 20 ms and 2 pulses for MyLa), aiming for the highest transfection efficiency with the minimum loss of cell viability. Cells were then electroporated under these conditions to allow for the entry of the siRNAs into the cell and 100 µl of the suspension were seeded in 2 ml for 24, 48, 72 and 96 h.

In addition, a stable PIM knockdown was carried out in PTCL cell lines using the MISSION product line from Sigma-Aldrich (St Louis, MO) according to the manufacturer’s instructions. The base vector (pLKO.1-puro) contains the Puromycin resistance gene for mammalian cells selection. Thus, sensitivity to Puromycin was first tested in several PTCL cell lines, and optimal concentrations were chosen from a wide range. Then, the optimal amount of lentiviral particles was assessed using the control transduction particles, both the negative (Non-targeting shRNA, SHC016V) and the positive (Turbo-GFP, SHC003V) lentiviruses (Sigma-Aldrich). MyLa was the only used cell line showing high infection efficiency, and therefore, was the best model to test PIM stable knockdown. Briefly, MyLa cells were infected with MISSION lentiviral transduction particles containing specific short-hairpin RNA (shRNA) against *PIM1* (SHCLNV-NM_002648), *PIM2* (SHCLNV-NM_006875) and *PIM3* (SHCLNV-NM_001001852) (all from Sigma-Aldrich) using Polybrene (hexadimethrine bromide) as a transduction enhancer (8 µg/ml). After 24 h post-infection, lentiviral particles were removed and Puromycin (4 µg/ml) was added to culture media. Green fluorescence was checked for 15 days using a Nikon Ti Epi-Fluorescence microscope and the imaging software NIS-Elements (Nikon, Amsterdam, Netherlands) and flow cytometry.

### Cell viability assay

For drug cytotoxicity experiments, PTCL cell lines and primary tumoral and normal T cells were seeded in 96-well plates at a density of 10,000 cells per well, and pan-PIM inhibitors, ALKi, Cisplatin or combinations were added at a range of doses for 48 h (for primary cells) and 72 h (for cell lines), using DMSO as control. Cell viability was measured as the intracellular ATP content using CellTiter-Glo Luminescent Cell Viability Assay (Promega, Madison, WI), following the manufacturer’s instructions.

For drug combination experiments, cells were treated with a wide range of doses and cell viability was measured as explained above. The combination index (CI) was calculated using CalcuSyn software (Biosoft, Ferguson, MO) following the Chou & Talalay method [24], where values of CI<1, ≈1 and >1 indicate synergism, an additive effect and antagonism, respectively.

### Flow cytometry analysis

The distribution of cells during the phases of the cell cycle and induction of apoptosis were evaluated by flow cytometry using propidium iodide (PI, Sigma-Aldrich, St Louis, MO) staining and the APC-Annexin V (Beckton Dickinson, BD, Franklin Lakes, NJ) binding assay, respectively. Data from 10,000 cells were detected on a FACS Calibur flow cytometer (BD) and analyzed using CellQuest Pro software (BD).

### RNA extraction and quantitative RT-PCR

Total RNA was extracted and purified using RNeasy Mini-Kit (Qiagen, Valencia, CA) following the manufacturer’s instructions in order to check the PIM genes’ knockdown efficiency, to understand the molecular response to the pan-PIMi and to measure basal mRNA levels of these genes in PTCL cell lines and primary tumoral and normal T cells.

The expression of *PIM1, PIM2, PIM3, ERCC8, XRCC2* and *XRCC5* genes was measured by quantitative RT-PCR. Briefly, total RNA was retrotranscribed using the SuperScript enzyme (Life Technologies) (10 min at 25°C, 60 min at 42°C and 15 min at 70°C). Two µl of the resulting cDNA were placed in a 384-well plate with 0.75 µl Taqman probes (*PIM1* Hs01065494_g1, *PIM2* Hs00179139_m1, *PIM3* Hs00420511_g1, *ERCC8* Hs00163958_m1, *XRCC2* Hs03044154_m1, *XRCC5* Hs00897854_m1, and the endogenous control *RN18S1* Hs03928990_g1; all from Life Technologies) in a final volume of 15 µl. PCR amplification was performed using the Applied Biosystems Prism 7900HT Sequence Detection System (Life Technologies) under the following thermal cycler conditions: 2 min at 50°C, 10 min at 95°C and 30 cycles (15 s at 95°C and 1 min at 60°C).

Relative Quantification (RQ) was calculated following the ΔCt method: RQ = 2^−ΔCt^, where ΔCt is the difference between the Ct of the gene of interest and the Ct of the endogenous gene control *RN18S1.* In addition, in knockdown experiments RQ was normalized as RQ = 2^−ΔΔCt^, where ΔΔCt is the difference between the ΔCt in knockdown cells and the ΔCt in control cells.

### Microarray hybridization and data analysis

The molecular response of PTCL cells to the pan-PIMi ETP-39010 was explored through gene expression analysis. DERL7, HuT78, MyLa and SR786 cells were treated with 10 µM pan-PIMi for 0, 2, 4, 6, 10 and 24 h. At each time, DMSO- and pan-PIMi-treated cells were harvested and total RNA extracted as described above, and the quality assessed in a 1% agarose-gel. Samples were hybridized onto 4×44 K microarray slides (Whole Human Genome, Agilent Technologies, Inc., Santa Clara, CA), as described in the Supplementary Information (Methods S1). Short Time-series Expression Miner (STEM) [25] identified differentially expressed genes under each condition, and Gene Ontology (GO) categories were used to recognize functional groups. A False Discovery Rate (FDR) <0.05 was considered significant. All raw microarray data regarding the molecular response to pan-PIMi in PTCL are available at the Gene Expression Omnibus under accession number GSE42595.

### Immunofluorescence and immunohistochemistry

After treatment with 10 µM of the pan-PIMi ETP-39010 for 24 h, γH2A.X was measured in MyLa cells by immunofluorescence. Immunohistochemical staining of PIM2 was carried out in a series of formalin-fixed and paraffin-embedded tumoral samples from 136 PTCL patients (Table S1) in a Bond Max automatic immunostainer (Leica Microsystems, Wetzlar, Germany). Details are provided in the Supplementary Information (Methods S1).

### Statistical analysis

Unless otherwise specified, all experiments were done three times and all numerical data were expressed as the average of the values ± the standard error of the mean. Statistical significance of differences between groups was established by Student’s independent samples t-test (SPSS v17.0). p-values <0.05 were considered significant.

## Results

### PIM kinases as potential therapeutic targets in PTCL

**Figure 1:**
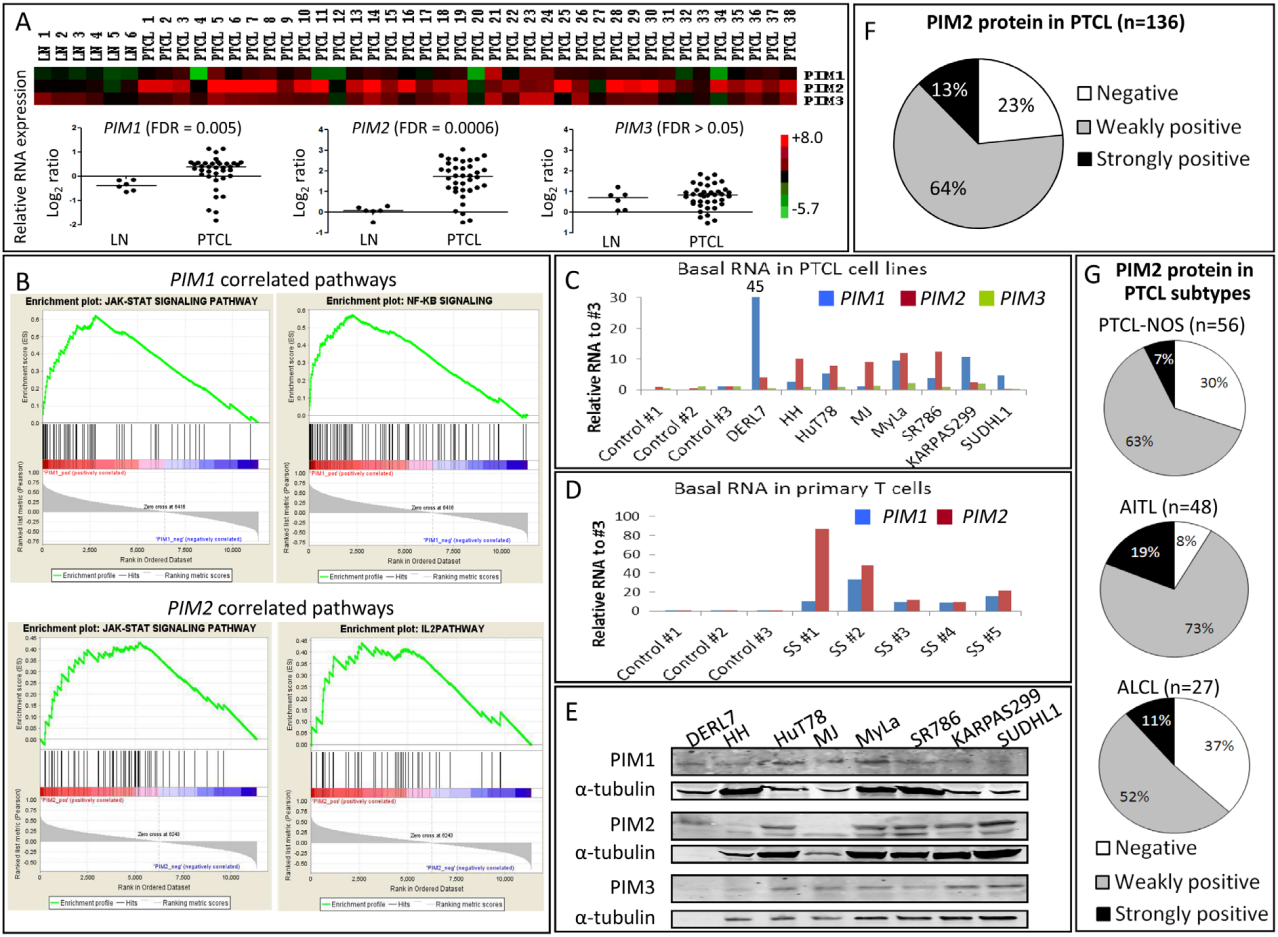
PIM kinases as potential therapeutic targets in PTCL. (A) Gene expression profiling of tumoral samples from 38 human PTCL patients compared with 6 reactive lymph nodes (LN) by microarrays revealed a significantly increased expression of *PIM1* and *PIM2* genes (FDR<0.05), but not *PIM3*. The heatmap is shown in the upper panel, and the relative quantification (Log_2_ ratio) comparing PIM expression in PTCL *versus* LN is shown in the lower panel. (B) GSEA ranked all significantly altered genes between PTCL and LN according to its correlation with *PIM1* or *PIM2* expression and displayed them in the red-to-blue bar. Each gene belonging to every pathway was interrogated whether it appeared positively (in the red region of the bar) or negatively (in the blue side) correlated. Using this approach GSEA identified a positive and significant correlation between *PIM1* and *PIM2* expression and Jak/STAT, NF-κB and IL-2 signaling pathways in the PTCL molecular signature (FDR<0.25). (C) PIM family genes mRNA level was measured by RT-qPCR in eight PTCL cell lines and (D) primary tumoral T cells from 5 Sézary Syndrome patients (SS #1–5), and compared with normal T cells isolated from 3 healthy donors (Control #1–3). The relative RNA amount of PIM has been calculated as a relative quantification, as described in the Methods section (RQ = 2^−ΔCt^), normalized with non-tumoral cells: RQ in PTCL/RQ in healthy #3. In both settings, *PIM1*, and especially *PIM2*, but not *PIM3* expression was found to be increased in PTCL. (E) PIM kinase protein basal levels in PTCL cell lines measured by Western blot. PIM1 and PIM2 isoforms are also shown. (F) Distribution of PIM2 protein in a series of tumoral samples from 136 PTCL patients measured by immunohistochemistry. Negative, weakly positive and strongly positive samples were defined by the presence of <5%, 5–20% and >20% positive cells. (G) Distribution of PIM2 protein in the most common PTCL subtypes measured by immunohistochemistry.

First, using microarrays, *PIM1* and especially *PIM2* genes, but not *PIM3*, were found to be significantly overexpressed (FDR<0.05) in tumoral samples from 38 PTCL patients compared with 6 reactive lymph nodes (Figure 1A). *PIM1* and *PIM2* expression was significantly correlated with Jak/STAT, NF-κB and IL-2 pathways in our PTCL patient series (Figure 1B), indicating a strong relationship between these pathways and the expression of PIM kinases in PTCL. Furthermore, *PIM1* and, again, especially *PIM2*, but not *PIM3* expression was increased in a panel of 8 PTCL cell lines (Figure 1C) and primary tumoral T cells from 5 Sézary Syndrome patients (Figure 1D) relative to normal T cells from 3 healthy donors. Similarly, PIM protein levels were also detected by Western blot in all PTCL cell lines, with slight differences in the most expressed PIM2 isoform (Figure 1E).

Since *PIM2* was the most upregulated PIM kinase in PTCL (both patients and cell lines) at the mRNA and protein levels, we explored the expression of the PIM2 protein by immunohistochemistry in a series of 136 PTCL patients. We found that 77% of these samples were positive for PIM2 expression (Figure 1F and Figure S1), and that the trend was largely maintained in the most common PTCL subtypes, with a slight predominance in the AITL subtype (Figure 1G). Although our series was limited, a preliminary significant association was found between PIM2 expression and a shorter overall survival only in the ALCL subtype, both ALK+ and ALK− cases, but not in other PTCL subtypes (Figure S2).

These findings suggest that PIM kinases could be of potential therapeutic value in PTCL.

### PIM kinase genetic silencing in PTCL

**Figure 2:**
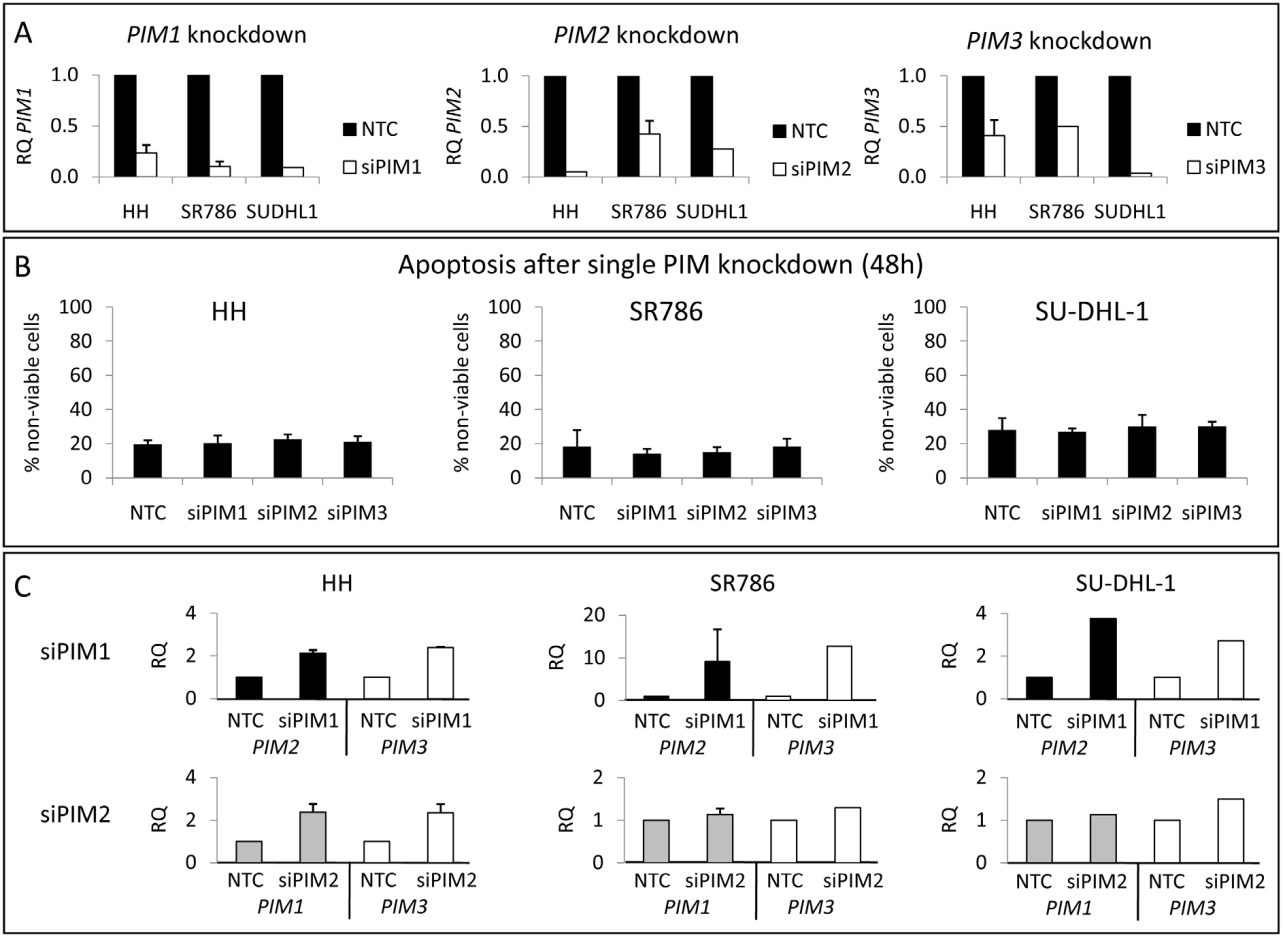
Individual *PIM1, PIM2* and *PIM3* genetic silencing in PTCL cell lines by siRNA. (A) All three PIM family genes were silenced in HH, SR786 and SU-DHL-1 cell lines, using several siRNA doses (25–100 nM) and times (24–72 h). Knockdown efficiency was measured by RT-qPCR and compared with the Non-Template Control (NTC). Graphs show the silencing 24 h after the addition of 100 nM siRNA. RQ, relative quantification, has been calculated as described in the Methods section (RQ = 2^−ΔΔCt^). (B) Single PIM gene silencing did not induce apoptosis in any PTCL cell lines. Graphs show data from 48 h silencing. The percentage of non-viable cells was calculated as Annexin V+/PI- plus Annexin V+/PI+ cells. (C) RT-qPCR showing a compensatory mechanism among the PIM family genes: when one PIM gene was silenced, the other PIM genes became upregulated. RQ, relative quantification, has been calculated as described in the Methods section (RQ = 2^−ΔΔCt^).

To test this hypothesis, genetic silencing experiments with siRNAs were performed specifically to abolish the expression of *PIM1, PIM2* or *PIM3* genes in PTCL cell lines. Knockdown efficiency differed with the cell line and time point, varying from around 70 to 95% in the *PIM1* or *PIM2* mRNA, and lower for *PIM3* (Figure 2A). However, no significant effects on cell survival were observed, either with respect to apoptosis induction (Figure 2B and Table S2) or cell cycle arrest (Figure S3). These results indicated that the remaining protein or other untargeted genes were responsible for triggering survival.

As PIM genes belong to the same family and have a high homology in their sequences [15], [19], [20], they could share functions. This prompted us to measure the mRNA levels of each of the PIM members when one of them was knocked down. Strikingly, we found an upregulation of *PIM2* and *PIM3* when *PIM1* was silenced. Likewise, an increase in *PIM1* and *PIM3* was observed when *PIM2* was inhibited (Figure 2C). Because the *PIM3* knockdown was less efficient than other PIM genes silencing, *PIM1* or *PIM2* upregulation after *PIM3* inhibition was less evident (data not shown). Again, very similar results were found in all cell lines, suggesting the existence of a compensatory mechanism among the PIM genes in PTCL.

**Figure 3:**
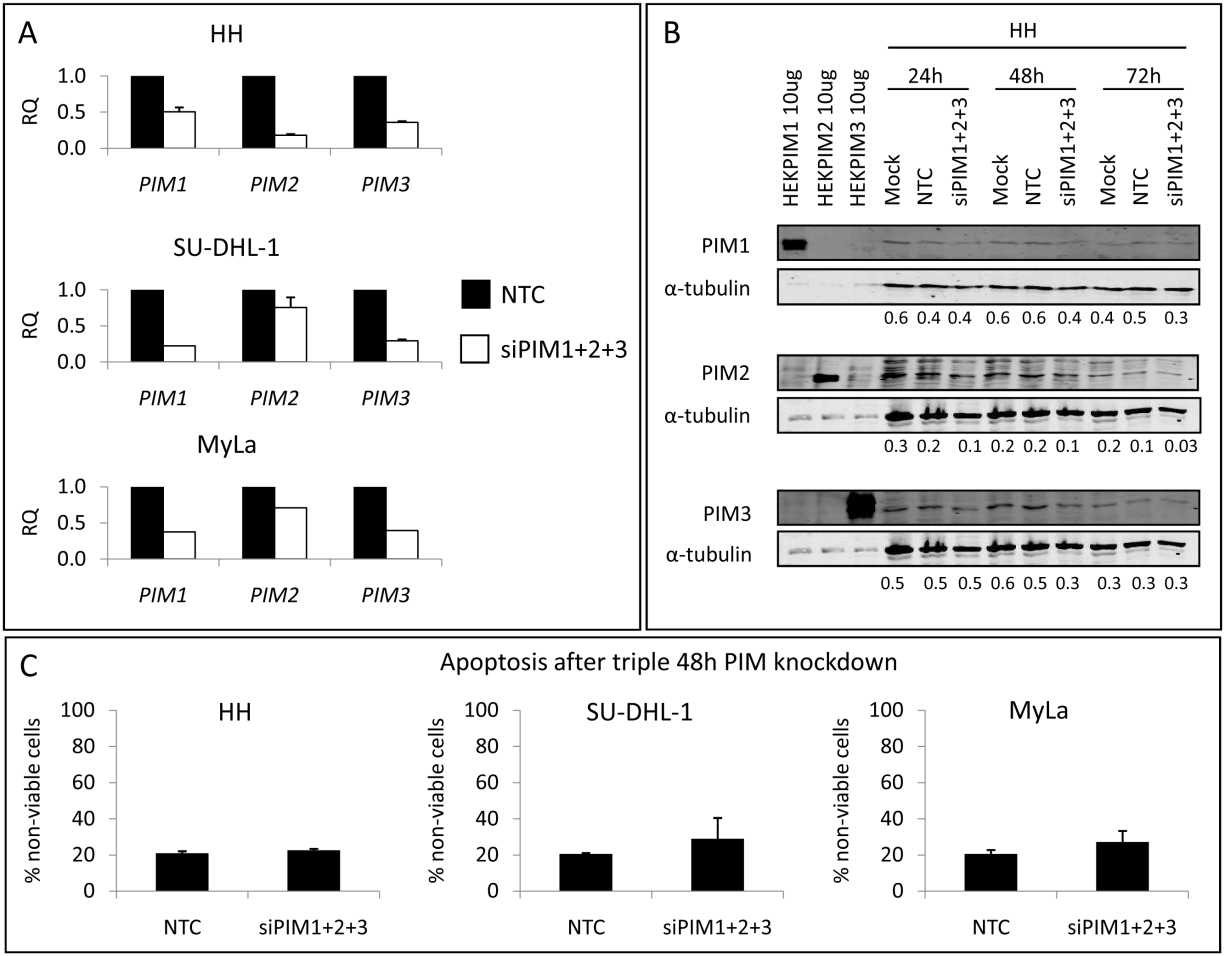
Simultaneous *PIM1+ PIM2+ PIM3* genetic silencing in PTCL cell lines by siRNA. (A) The triple knockdown was carried out in the HH, SU-DHL-1 and MyLa cell lines, using 33 nM siPIM1+33 nM siPIM2+33 nM siPIM3 for 24–72 h. As an average for the 3 PIM genes, the knockdown efficiency, measured by RT-qPCR, was around 70% at the mRNA level 24 h after the microporation (RQ, relative quantification, calculated as in Figure 2), and (B) around 50% at the protein level after 48 h, compared with the Non-Template Control (NTC). Numbers presented are PIM/tubulin ratios. (C) These knockdown conditions did not induce apoptosis in any PTCL cell lines. The percentage of non-viable cells was calculated as Annexin V+/PI− plus Annexin V+/PI+ cells.

This led us to exploit the simultaneous silencing of the 3 PIM genes: since the recommended maximum siRNA concentration is 100 nM and we aimed to inhibit 3 genes at the same time, the concentration of each siRNA was reduced to 33 nM. This meant that the knockdown efficiency was lower than for individual silencing: around 70% at the mRNA level on average for the 3 genes 24 h after the microporation (Figure 3A), and about 50% at the protein level after 48 h (Figure 3B). Once more, however, cell survival was unaffected, producing no significant induction of apoptosis (Figure 3C) or cell cycle arrest (Figure S4) in any cell line under any of the studied conditions. These results could be due to the transient knockdown triggered by siRNAs.

**Figure 4:**
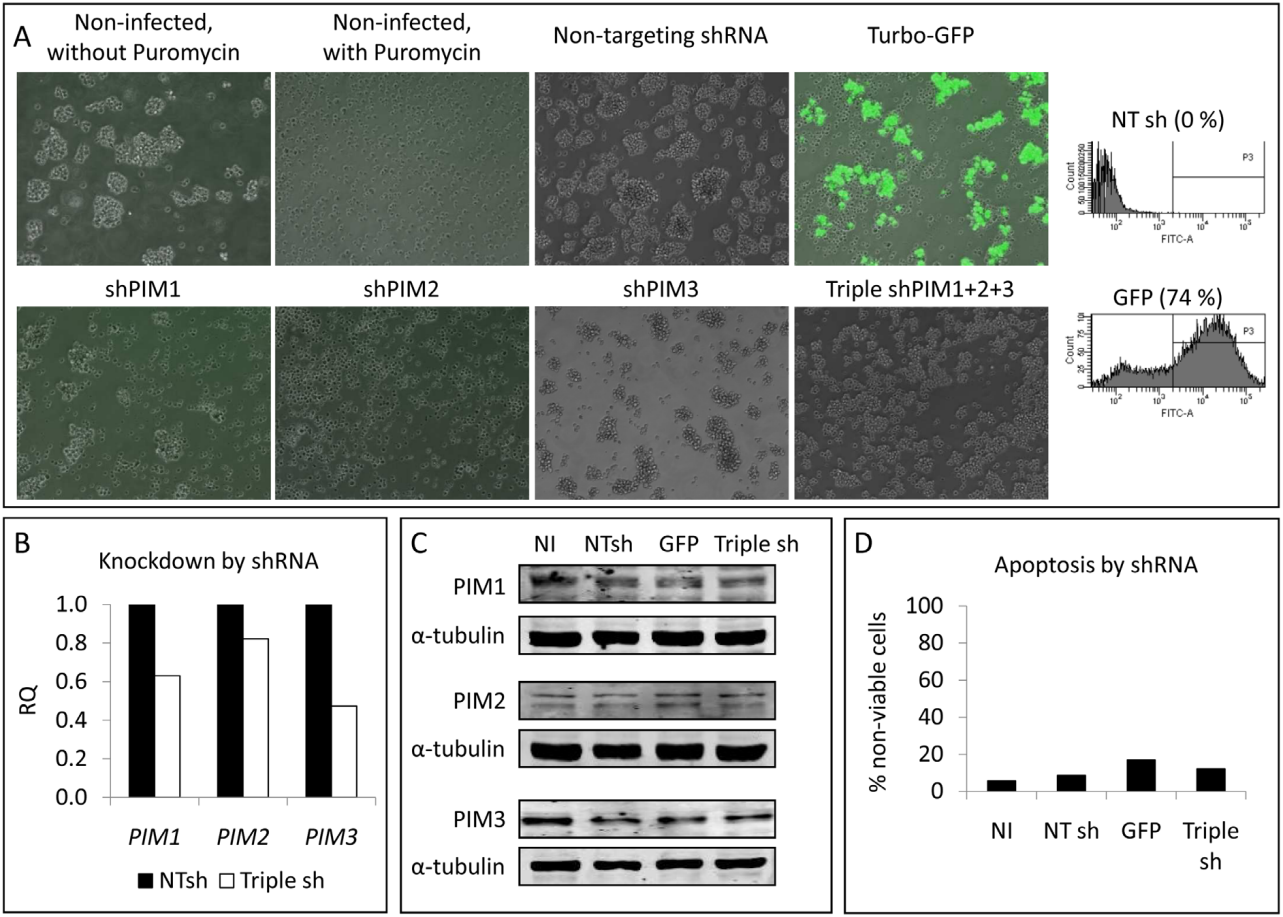
PIM genetic silencing in PTCL by shRNA. (A) MyLa cells were infected with lentiviral particles containing the pLKO.1-puro vector with a non-targeting shRNA, the Turbo-GFP gene, or shRNAs for each of the PIM family genes. Cells were maintained under Puromycin selection for 3 weeks. Images were obtained 8 days post-infection with a Nikon Ti Epi-Fluorescence microscope (10X magnification). Green fluorescence was also assessed in the negative (NT sh) and the positive controls by flow cytometry, and percentages of green cells are indicated in the histograms. (B) Triple knockdown efficiency was measured by RT-qPCR and compared with the Non-Targeting shRNA (NTsh). Graphs show the silencing at the mRNA level 15 days after the lentiviral infection. RQ, relative quantification, has been calculated as described in the Methods section (RQ = 2^−ΔΔCt^). (C) Western blots showing the effect of the triple lentiviral infection on PIM protein levels 15 days post-infection (NI, non-infected cells; NTsh, cells infected with the non-targeting shRNA; GFP, cells infected with Turbo-GFP; Triple sh, cells infected with shPIM1+ shPIM2+ shPIM3). (D) These knockdown conditions did not affect cell viability. The percentage of non-viable cells was calculated as Annexin V+/7AAD− plus Annexin V+/7AAD+ cells: (NI, non-infected cells; NTsh, cells infected with the non-targeting shRNA; GFP, cells infected with Turbo-GFP; Triple sh, cells infected with shPIM1+ shPIM2+ shPIM3).

Additionally, a more stable knockdown of PIM genes was approached using lentiviral particles containing shRNAs inserted into the pLKO.1-puro vector. Since infected cells should be selected with Puromycin, first we tested the sensitivity of several PTCL cell lines to this antibiotic. SU-DHL-1 and SR786 cell viability was rapidly impaired in the presence of Puromycin, while HH and MyLa cells showed a greater resistance (data not shown). Then, we checked the infection efficiency using lentiviral particles containing GFP, and observed a high proportion of green MyLa cells, while no green HH cells were found even after 15 days post-infection (data not shown). Thus, MyLa cell line was chosen to be infected with shPIM-lentiviral particles: 74% of cells were infected 8 days after lentiviral addition (Figure 4A). This efficiency was checked to be as high as possible, because in parallel, non-infected cells were cultured in the presence of Puromycin, and at this time point all these cells were dead (Figure 4A). However, under these conditions around 30% of PIM-mRNA was still detectable by RT-qPCR (data not shown). Even when the 3 shRNAs (shPIM1+ shPIM2+ shPIM3) were simultaneously added and cells were selected for 15 days, we found a decrease of only 30%, on average, both at the mRNA (Figure 4B) and protein (Figure 4C) levels. Again, these conditions did not affect cell survival (Figures 4A and 4D).

These results could be due to the incomplete silencing of all 3 PIM kinases, with the remaining active protein still triggering enough survival signaling.

### Pharmacological pan-PIM kinase inhibition in PTCL

**Figure 5:**
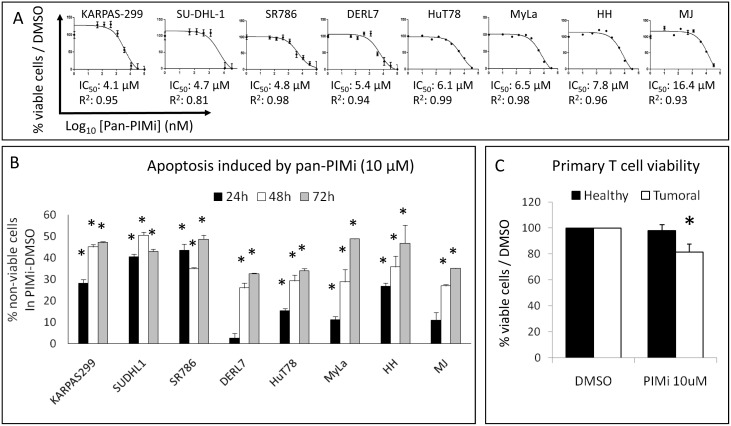
Pharmacological pan-PIM kinase inhibition in PTCL cell lines. (A) The pan-PIMi ETP-39010 reduced cell viability in all PTCL cell lines (IC_50_ values calculated after 72 h of treatment are shown). (B) The pan-PIMi ETP-39010 strongly induced apoptosis in a time-dependent manner in all PTCL cell lines (*, p<0.05, from comparison with DMSO-treated cells). The percentage of non-viable cells was calculated as Annexin V+/PI− plus Annexin V+/PI+ cells in the PIMi-treated condition minus the DMSO-treated control. (C) The pan-PIMi ETP-39010 (10 µM for 48 h) slightly but significantly reduced cell viability only in tumoral T cells from 8 PTCL patients (Mycosis Fungoides and Sézary Syndrome), but did not affect normal T cells from 5 healthy donors (*, p<0.05 compared with DMSO).

In order to inhibit the catalytic activity of all PIM kinases more efficiently, the pharmacological pan-PIM inhibitor ETP-39010 [21] was used. We found that this drug reduced cell viability in all PTCL cell lines in the same low micromolar range of IC_50_ values (Figure 5A). This effect was mainly due to a strong dose- and time-dependent induction of apoptosis (Figure 5B and Figure S5A, S5B and S5C), without cell cycle arrest (Figure S5D). The subG0 population (reflecting dead cells) increased even with short-duration, low-dose treatments, especially in the KARPAS-299, SU-DHL-1 and SR786 cell lines, which are members of the ALCL subtype (Figure S5D).

These results indicated a direct and strong cytotoxic effect of the pharmacological pan-PIMi on PTCL cell lines.

Additionally, we tested the *ex vivo* efficiency of the pan-PIMi in PTCL. Primary T cells from 8 PTCL patients (Mycosis Fungoides and Sézary Syndrome) and 5 healthy donors were treated for 48 h, and we observed that tumoral T cell viability was slightly but significantly reduced, while normal T cells remained unaffected (Figure 5C).

### Molecular response of PTCL to the pharmacological pan-PIMi

**Figure 6:**
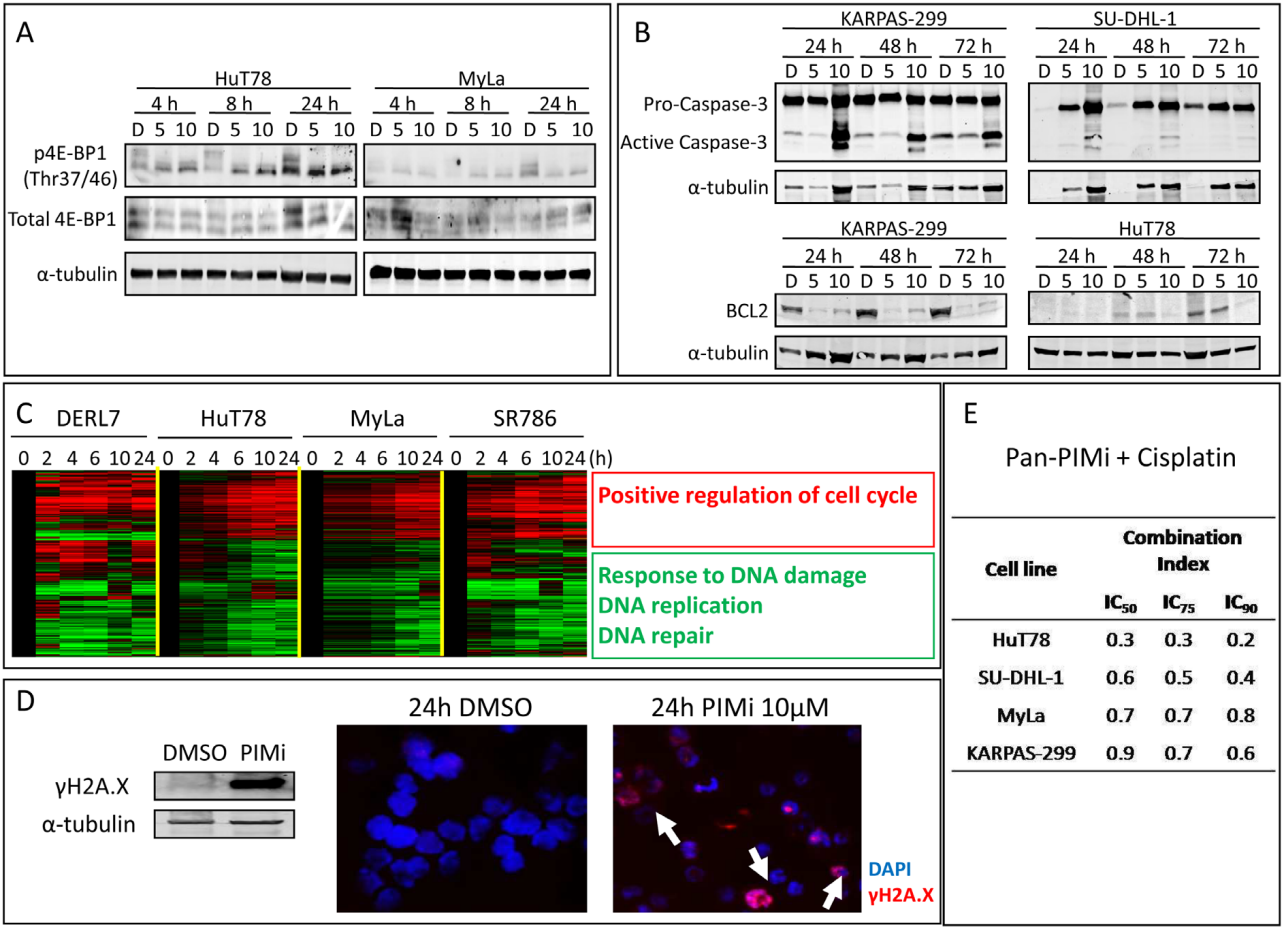
Molecular response to the pan-PIMi in PTCL. (A) The pan-PIMi ETP-39010 reduced phosphorylation of 4E-BP1 in PTCL cell lines. D: DMSO; 5: 5 µM pan-PIMi; 10: 10 µM pan-PIMi. (B) Key effectors of apoptosis, such as Caspase-3 and BCL2, were affected by the pan-PIMi in PTCL cell lines, in a time and dose-dependent manner. D: DMSO; 5: 5 µM pan-PIMi; 10: 10 µM pan-PIMi. (C) Heat-map showing the commonly differentially expressed genes (FDR<0.05) in all PIMi-treated cell lines (10 µM) compared with DMSO-treated cells at each time point. STEM program was used to identify significant genes, and FatiGO recognized the pathways in which they were involved (adjusted p-value<0.05). (D) Amount and pattern of distribution of γH2A.X was tested by Western blot and immunofluorescence, respectively, in MyLa cells treated with 10 µM pan-PIMi for 24 h. Arrows show γH2A.X foci (40X magnification). Images were obtained by a fluorescence microscope (Axio Imager Z1, Zeiss, Oberkochen, Germany). (E) PTCL cell lines were treated with a range of doses of the combination pan-PIMi + Cisplatin for 72 h. Combination Index (<1) showed synergism between both drugs in all studied PTCL cell lines.

To confirm that the pharmacological pan-PIMi was really inhibiting PIM kinase activity, we measured the phosphorylation status of 4E-BP1, a well-established substrate of PIM kinases [13], [14], [15]. A decrease in p4E-BP1 was found in PTCL cell lines after short treatment with pan-PIMi (Figure 6A). Moreover, taking into account the dramatic proapoptotic effect of this drug, two key proteins involved in apoptosis were also examined: we found that the pan-PIMi induced cleavage and activation of Caspase-3 and decreased the levels of BCL2 (Figure 6B). These observations support and explain the strong apoptosis induced by the pan-PIMi in PTCL.

To understand the molecular response of PTCL cells to the pharmacological pan-PIMi, 4 PTCL cell lines were treated with 10 µM for varying periods, and changes in gene expression over time were examined. We found 390 genes that were differentially expressed (FDR<0.05) and commonly deregulated in all 4 cell lines upon drug treatment (Figure 6C). On the basis of GO categories we found that the upregulated genes were those involved in the positive regulation of the cell cycle pathway, which could explain the aforementioned absence of cell cycle arrest, and that the downregulated genes were related to the response to DNA damage, repair and replication, which could be added and enhance the strong apoptosis induced by the pan-PIMi (Figure 6C). A more detailed list of genes and pathways deregulated in each PTCL cell line treated with the pan-PIMi can be found in the Tables S3 and S4, respectively.

To validate this result, the expression of several genes involved in DNA damage repair, such as *ERCC8, XRCC2* and *XRCC5* (Figure S6A) was measured by RT-qPCR. We found that treatment with the pan-PIMi downregulated the expression of these genes in a time- and dose-dependent manner (Figure S6B).

To functionally confirm that pharmacological PIM kinase inhibition impaired the DNA damage repair machinery, we measured the amount and distribution of γH2A.X protein, the classical hallmark for DNA damage [26], [27], in MyLa cells. After treatment with the pan-PIMi increases in the amount and formation of γH2A.X foci corresponding to DNA damage foci were observed (Figure 6D).

These results indicated that the pharmacological pan-PIMi strongly induces DNA damage through the downregulation of genes involved in the DNA repair machinery.

Based on this, we hypothesized that the response to the pan-PIMi could be even improved by a DNA damaging agent, such as Cisplatin. Thus, 4 PTCL cell lines were treated with the drug combination pan-PIMi + Cisplatin for 72 h. In all tested cell lines, a synergistic effect between both drugs (Combination Index <1) was observed (Figure 6E), highlighting again the functional link between PIM kinases and DNA repair.

### Synergism between PIM and ALK inhibition in ALCL

**Figure 7:**
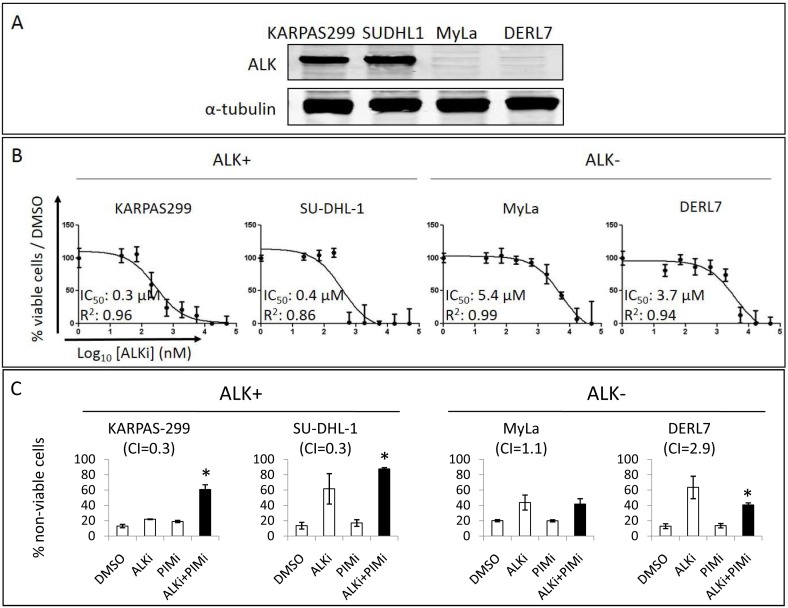
Synergism between ALK and PIM inhibition in ALCL. (A) ALK expression was explored by Western blot in 4 PTCL cell lines. (B) IC_50_ values were measured upon 72 h treatment with the ALKi Crizotinib: ALK+ ALCL cell lines were around 10 times as sensitive to the ALKi as the ALK− cells. (C) Cells were treated for 24 h with IC_50_ of ALKi and pan-PIMi, alone and combined. The combination of ALKi + PIMi was highly synergistic (Combination Index, CI<1) and strongly enhanced apoptosis in ALK+ ALCL cell lines after 24 h, while this combination was antagonistic in ALK− PTCL cell lines (CI>1) (*, p<0.05 in comparison with DMSO). Data represent Annexin V+/PI− and Annexin V+/PI+ cells in each treatment. Black columns highlight the combined treatment.

Since ALCL cell lines were the most sensitive to the pan-PIMi ETP-39010, and PIM2 expression was preliminarily associated with poor prognosis in our limited ALCL series, we decided to explore the therapeutic relevance of the PIM pathway in ALCL, especially in the ALK+ ALCL subtype, because ALK translocation is known to activate STAT3 [5], [6], and STAT3 triggers PIM2 expression [13], [14]. First, we treated 2 ALK+ ALCL cell lines (KARPAS-299 and SU-DHL-1) and 2 ALK− PTCL cell lines (MyLa and DERL7) with the ALKi Crizotinib (Figure 7A) and found, as expected, that the ALK+ cells were about 10 times as sensitive as the ALK− cells (Figure 7B). Next, we combined this ALKi with the pan-PIMi ETP-39010 to inhibit the same pathway at two sites, which produced a strong synergistic effect between these drugs only in ALK+ cells, but not in ALK− cells. Strikingly, 24 h of the combined treatment strongly enhanced apoptosis in KARPAS-299 and SU-DHL-1 cells (Combination Index <1), while a slight additive effect or even antagonism, was found in MyLa and DERL7 cells (Combination Index >1) (Figure 7C). The effects driven by the pan-PIMi shown here (24 h, 5 µM) were comparable with those in Figure S5A.

**Figure 8:**
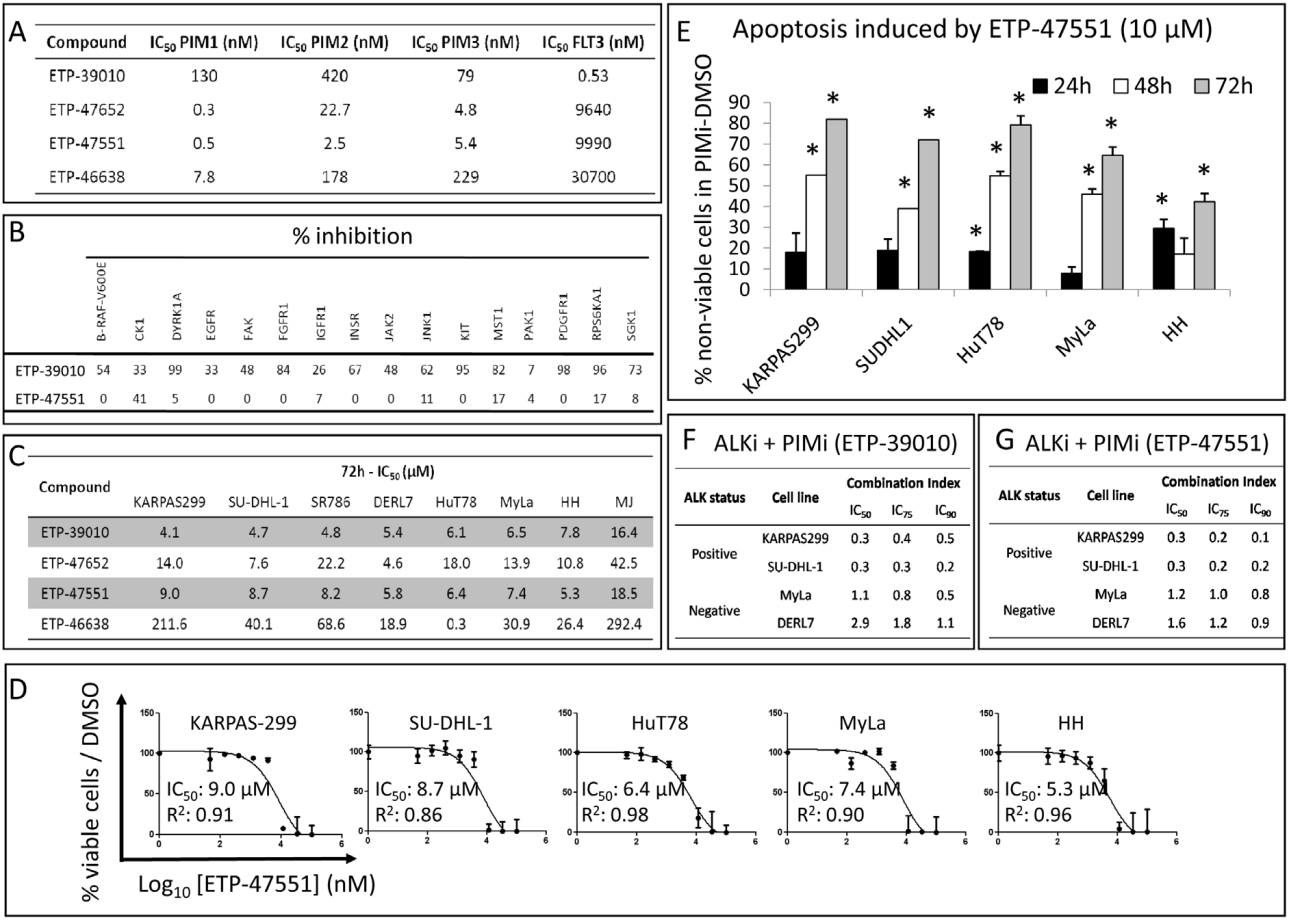
Comparison between the compound ETP-39010 and other pan-PIMi. (A) Selectivity profile showing the IC_50_ values of each of the compounds for the kinase activity of the indicated enzymes. (B) Percentage of inhibition of a panel of unrelated kinases by ETP-39010 and ETP-47551. A similar profile was found for ETP-47551, ETP-47652 and ETP-46638 compounds. (C) Sensitivity of PTCL cell lines to all pan-PIMi. (D) The newly developed pan-PIMi ETP-47551 reduced cell viability in all studied PTCL cell lines (IC_50_ values calculated after 72 h of treatment are shown). (E) The pan-PIMi ETP-47551 strongly induced apoptosis in a time-dependent manner in all studied PTCL cell lines (*, p<0.05, from comparison with DMSO-treated cells). The percentage of non-viable cells was calculated as Annexin V+/7AAD− plus Annexin V+/7AAD+ cells in the PIMi-treated condition minus the DMSO-treated control. (F) The combination of ALKi + ETP-39010 was highly synergistic only in ALK+ ALCL cell lines, as was (G) the combination of ALKi + ETP-47551 (Combination Index, CI, <1 indicates synergism between the two drugs; CI ≈1 indicates an additive effect; CI>1 indicates antagonism).

Finally, since the selectivity profile of the pan-PIMi ETP-39010 was not very specific, it was important to rule out the possibility that the effects we observed were due to off-target consequences. To this end, we confirmed the most significant results with newly developed and more specific pan-PIMi: ETP-47652, ETP-47551 and ETP-46638. Of these, ETP-47551 was the compound with the best selective profile (Figures 8A and 8B). We treated our panel of 8 PTCL cell lines with these new pan-PIMi, and found that all cell lines showed the highest sensitivity to the ETP-47551, with IC_50_ values comparable to those obtained with ETP-39010 (Figure 8C). Additionally, we observed that the more specific ETP-47551 reduced cell viability (Figure 8D) and strongly induced apoptosis in PTCL cell lines in a time-dependent manner (Figure 8E), similarly to the ETP-39010 compound. Lastly, the synergistic effect between the ALKi and the pan-PIMi ETP-39010 in ALK+ ALCL (Figure 8F) was confirmed using the combination ALKi plus the specific pan-PIMi ETP-47551 (Figure 8G). These results could help discard potential off-target effects driven by ETP-39010.

## Discussion

We hypothesized that PIM kinase inhibition could be of therapeutic value in PTCL because: 1) PIM kinases have an important role in CD4+ T cell responses [28]; 2) *PIM1* and especially *PIM2* expression is increased in PTCL patients, cell lines and primary tumoral T cells of Sézary Syndrome patients; 3) they are significantly correlated with survival pathways, such as Jak/STAT, NF-κB and IL-2 signaling; and 4) pharmacological PIM inhibition is effective in other T cell-mediated malignancies, such as T cell acute lymphoblastic lymphoma [29].

PIM family members, especially *PIM2*, were found to be overexpressed in PTCL, as in many other tumor entities of hematological or epithelial origin, such as chronic lymphocytic leukemia, mantle cell lymphoma, diffuse large B cell lymphoma, acute myeloid leukemia, and prostate, pancreatic, gastric, colon and hepatocellular carcinomas [13], [15]. This increased expression was found to be significantly correlated with Jak/STAT, NF-κB and IL-2 signalings in our PTCL patient series, suggesting that these pathways could be the responsible for PIM activation and could contribute to PTCL cell survival.

However, in spite of all of the supporting evidence, our results indicate that individual genetic silencing of *PIM1*, *PIM2* or *PIM3* genes does not affect PTCL cell survival, either at the level of apoptosis or the cell cycle. We found that this could be due, at least in part, to a compensatory mechanism among the 3 PIM genes, since *PIM1* knockdown was accompanied by the upregulation of both *PIM2* and *PIM3*, and vice versa. These redundant functions have also been described in *in vivo* models: mice lacking *PIM1* had a higher level of *PIM2* expression, while those deficient for *PIM1* and *PIM2* selectively activated *PIM3* [14], [15]. These overlapping functions can be explained by the substantial homology (50–70%) of the PIM kinases at the amino acid level [15], [19], [20], [30].

These observations suggested that in order to effectively treat PTCL an inhibition of all three PIMs would be required, as described for other hematological malignancies, such as multiple myeloma [31] and acute myeloid leukemia [32].

Unexpectedly, the simultaneous genetic inhibition of *PIM1+ PIM2+PIM3* did not affect PTCL cell survival either. Nevertheless, it is important to note that the simultaneous use of 3 siRNAs makes them less efficient than when they are on their own; moreover, these cells are not easily transfected/infected, making the genetic inhibition approach less informative. The lack of effect could be due to incomplete PIM family inhibition (indeed, around 50% of each protein remained after triple-knockdown si/shRNA, in our case), or the dispensable effects that have been described for PIM (the triple-knockout mice were still viable and their mainly described phenotypic characteristics was a markedly reduced body size [13], [14], [15]). To address this possibility, we adopted a pharmacological inhibition approach to abolish all PIM kinase activity. Although it is conceivable that the different ATP-binding region of PIM kinases compared with other kinases would allow specific PIM inhibitors to develop, in practice this specificity does not seem to be reached, especially because they also inhibit FLT3, PDGFR and KIT [15], [19], [21]. Some pharmacological inhibitors are available that are selective for one of the PIM kinases [21], but consequently they will not avoid the compensatory mechanism among the other PIM kinases. We used the ETP-39010 compound, which is a pan-PIMi with a low specificity profile [21] for all the functional assays. Nevertheless, inhibition of PIM kinases by this drug was assessed, since it reduced 4E-BP1 phosphorylation, which is a very well established PIM kinase substrate [13], [14], [15], [21], and could be a biomarker for PIM kinase inhibition in PTCL. In addition, the most significant effects observed with ETP-39010 were confirmed with a newly developed and much more selective compound (ETP-47551). Among our most striking findings was the potent cytotoxic effect in all PTCL cell lines upon pan-PIMi treatment, at doses similar to those used with other pan-PIMi, such as SGI-1776 in prostate cancer [33], acute myeloid leukemia [19], chronic lymphocytic leukemia [17] and mantle cell lymphoma [18]. Surprisingly, and in contrast to the findings of the great majority of these studies, apoptosis induction was not accompanied by cell cycle arrest in PTCL cell lines. An increase in the subG0 population was observed even at lower doses or with shorter-duration treatments, highlighting the potent efficiency of this pan-PIMi in PTCL, especially in ALCL cell lines. Moreover, this strong induction of apoptosis was in part due to the cleavage of Caspase-3, the decrease in BCL2 protein levels (as extensively described in [13], [14], [15], [32]), and the enhancement of the DNA damage, since we found that the pan-PIMi downregulated the expression of a number of genes involved in DNA damage repair signaling, leading to the formation of γH2A.X foci, the most well established surrogate biomarker for DNA damage [26], [27]. Accordingly, there are several lines of evidence involving PIM kinases in the DNA repair machinery [34], [35], [36], [37].

The cytotoxic effect found in pan-PIMi-treated PTCL cell lines was explored *ex vivo* in primary T cells from cutaneous T cell lymphoma patients. Interestingly, we found that although the effects on neoplastic cells was not very dramatic, normal T cells from healthy donors were not affected by the pan-PIMi, recalling the limited cytotoxicity observed in SGI-1776-treated normal lymphocytes [19]. These evidences could support the proof of concept that the PIM kinase inhibition strategy might be a preliminary safe therapeutic approach. Moreover, it has been reported that SGI-1776 treatment reduces tumor volume without causing significant changes in body weight [19]. These findings, along with the fact that the triple *PIM1+PIM2+PIM3* knockout mice had a mild phenotype [13], [14], support the rationale of using pharmacological pan-PIMi as safe antitumoral agents.

A large fraction of the PTCL patients showed increased PIM2 protein expression, regardless of their subtype (although with a slight predominance in AITL, where a PIM2 increased expression has been already reported [38]). Importantly, PIM2 protein levels were significantly correlated with a worse outcome in ALCL patients, as described for the majority of malignancies [13]. It is important to note that in our limited ALCL series (n = 27), this association between PIM2 expression and a worse survival was found taking into account both ALK+ and ALK− ALCL patients. Although more samples are needed if more statistically significant conclusions are to be drawn, since in our series, the well-known prognostic marker ALK expression is almost significantly associated with outcome (p = 0.08).

This preliminary finding, along with the fact that ALCL cell lines are the most sensitive to the pan-PIMi ETP-39010, led us to hypothesize that the ALK - STAT3 - PIM2 pathway could be important for ALCL survival, at least in ALK+ ALCL, since ALK is a well-known STAT3 activator [5], [6], and STAT3 has been extensively described to increase *PIM2* expression [13], [14]. Thus, we aimed to target this axis at two different points using the ALKi Crizotinib plus pan-PIMi (i.e., the less specific ETP-39010 and the more selective ETP-47551). As expected, the simultaneous inhibition of ALK and PIM strongly affected cell survival in ALK+ ALCL but not in other PTCL cell lines, synergizing the apoptosis induced by each drug alone only in ALK+ ALCL cells. These results could highlight the potential therapeutic usefulness of this pathway in ALK+ ALCL.

Although ALK+ ALCL is the PTCL subtype with the most favorable outcome, frequently relapses have been reported in around 30% of patients treated with primary chemotherapy [39]. Some studies have recently described the efficacy of ALK inhibition in ALCL, both in murine models [40] and in preliminary clinical studies: in one trial 2 ALK+ ALCL patients reported complete remission of the disease within 1 month of treatment with Crizotinib, the response being sustained 5–6 months later [8]. A later clinical trial with 9 ALK+ ALCL patients treated with Crizotinib showed an objective response rate of 100%, a complete remission rate of 100%, a median duration of response of 10 months and 3-year progression-free survival of 63% with a plateau in the curve after 6 months [9]. In fact, Crizotinib has been approved by the FDA for the treatment of ALK+ non-small cell lung cancer [3]. Unfortunately, despite its initially impressive efficacy, resistance to Crizotinib has been found in patients carrying mutations in the fused ALK proteins [9], [41]. For this reason, it might be worthwhile exploring drug combinations targeting downstream effectors of the oncogenic-driver ALK translocation. Additionally, PIMi are known to synergize strongly with other antitumoral agents, such as Cisplatin, as demonstrated here in PTCL, the MEK inhibitor UO126 in precursor T cell lymphoblastic leukemia [29], the PI3 K inhibitor GDC-0941 in acute myeloid leukemia [21], the HDAC inhibitor SAHA in classical Hodgkin lymphoma [42], Bendamustine in mantle cell lymphoma and splenic marginal zone lymphoma [43], the BCL2 inhibitor ABT-737 [44] and taxanes [45] in prostate cancer and the multi-kinase inhibitor Sunitinib in renal cell carcinoma [46].

In conclusion, our results suggest that the simultaneous inhibition of all PIM kinases could be an efficient therapeutic strategy in those PTCL with PIM upregulation. This strategy seems to be particularly relevant in the ALK+ ALCL subtype, whereby the increased expression of PIM2 is associated with shorter survival and the combinatory inhibition of ALK and all PIM kinases potently enhanced apoptosis.

## Supporting Information

Figure S1 **PIM2 protein in tumoral samples from PTCL patients.** Representative immunohistochemical stainings for PIM2 (A) negative (<5% positive cells), (B) weakly positive (5–20% positive cells) and (C) strongly positive (>20% positive cells) samples from PTCL patients, specifically, a PTCL-NOS and two AITL, respectively (upper panels at 20X magnification and lower panels at 100X magnification).
(TIF)

Figure S2 **Association between PIM2 protein expression and overall survival in PTCL patients.** PIM2 protein (both weak and strong signal) was significantly associated with worse overall survival in ALCL (n = 27), but not in the PTCL-NOS (n = 42) + AITL (n = 39) subgroups.
(TIF)

Figure S3 **Effects of single PIM genetic knockdown on cell cycle in PTCL cell lines.** Individual PIM gene inhibition (100 nM siRNA) did not induce cell cycle changes over the time. (NTC: non-template control).
(TIF)

Figure S4 **Effects of triple PIM genetic knockdown on cell cycle in PTCL cell lines.** Simultaneous triple *PIM1*+*PIM2*+*PIM3* gene inhibition did not induce cell cycle changes over the time. (NTC: non-template control).
(TIF)

Figure S5 **Effects of the pharmacological pan-PIMi on PTCL cell survival.** (A) PTCL cell lines were treated with 5 µM of pan-PIMi for 24–72 h and effects on apoptosis were measured by flow cytometry. The percentage of non-viable cells was calculated as Annexin V+/PI− plus Annexin V+/PI+ cells in the PIMi-treated condition minus the DMSO-treated control. The pan-PIMi ETP-39010 strongly induced apoptosis in a time-dependent manner in all PTCL cell lines (*, p<0.05, from comparison with DMSO-treated cells). (B) Original scatter plots from FACS characterizing the effect of the pharmacological pan-PIMi on apoptosis in ALK+ ALCL cell lines: the X axis represents Annexin V staining and the Y axis represents PI staining. Representative plots from 3 independent experiments. (C) Original scatter plots from FACS characterizing the effect of the pharmacological pan-PIMi on apoptosis in other PTCL cell lines: the X axis represents Annexin V staining and the Y axis represents PI staining. Representative plots from 3 independent experiments. (D) The pan-PIMi (24 h) did not promote cell cycle arrest at any phase, but a direct increase in the subG0 fraction, as indicated numerically (mean ± SEM), especially in ALK+ ALCL cell lines (KARPAS-299, SU-DHL-1 and SR786).
(PDF)

Figure S6 **Downregulation of DNA damage repair signaling by the pharmacological pan-PIMi.** (A) Heat-map showing an overall downregulation of genes involved in DNA damage repair machinery driven by the pharmacological pan-PIMi (10 µM at indicated times) in both MyLa and SR786 cell lines. These expression changes were significant (FDR<0.05), and extracted from Table S3. Some important genes, such as *ERCC8, XRCC2* and *XRCC5* (highlighted by arrows) were randomly selected to be validated. (B) Validation of microarray data by RT-qPCR. The expression of *ERCC8, XRCC2* and *XRCC5* genes was confirmed to be reduced in a time- and dose- dependent manner after pan-PIMi treatment in MyLa and SR786 cell lines. RQ, relative quantification, was calculated as described in the Methods section as RQ = 2^−ΔCt^.
(TIF)

Table S1 **Clinical characteristics of the series of PTCL patients used for immunohistochemical studies.** PIM2 protein expression was explored in 136 PTCL patients. (PTCL-NOS: peripheral T cell lymphoma not otherwise specified; AITL: angioimmunoblastic T cell lymphoma; ALCL: anaplastic large cell lymphoma; NK-T: natural killer T cell lymphoma; IPI: international prognostic index; PIT: prognostic index for peripheral T-cell lymphoma, unspecified; ECOG: Eastern Cooperative Oncology Group; LDH: lactate dehydrogenase).
(TIF)

Table S2 **Effects of single PIM genetic knockdown on apoptosis in PTCL cell lines.** Individual PIM gene inhibition did not induce apoptosis over the time. The percentage of non-viable cells was calculated as Annexin V+/PI− plus Annexin V+/PI+ cells. (NTC: non-template control).
(TIF)

Table S3 **Significantly PIMi-deregulated genes in PTCL cell lines.** Differentially expressed genes in each cell line upon pan-PIMi treatment (10 µM) were identified using STEM program, which compared the expression profile in pan-PIMi-treated cells with DMSO-treated cells at each time point (0, 2, 4, 6, 10 and 24 h). Almost 400 genes were found significantly deregulated (FDR<0.05) upon pan-PIMi treatment. Expression values (log_2_ ratio) were normalized with the time point 0 h.
(XLS)

Table S4 **Significantly PIMi-deregulated pathways in PTCL cell lines.** Differentially expressed genes in each cell line upon pan-PIMi treatment identified by STEM (FDR<0.05) were applied to FatiGO to look for their functions. Significant biological processes at level 6 are shown (numbers indicate adjusted p-values). Red, green and white colors represent upregulation, downregulation and no significant deregulation, respectively. DNA-related processes are highlighted with arrows.
(TIF)

Methods S1 **Additional detailed methodology.**
(DOC)
