# Supplementary figures and images for: PIM Kinases as Potential Therapeutic Targets in a Subset of Peripheral T Cell Lymphoma Cases
Source: PLoS One. 2014 Nov 11;9(11):e112148. doi: 10.1371/journal.pone.0112148 (PMC4227704; doi:10.1371/journal.pone.0112148)

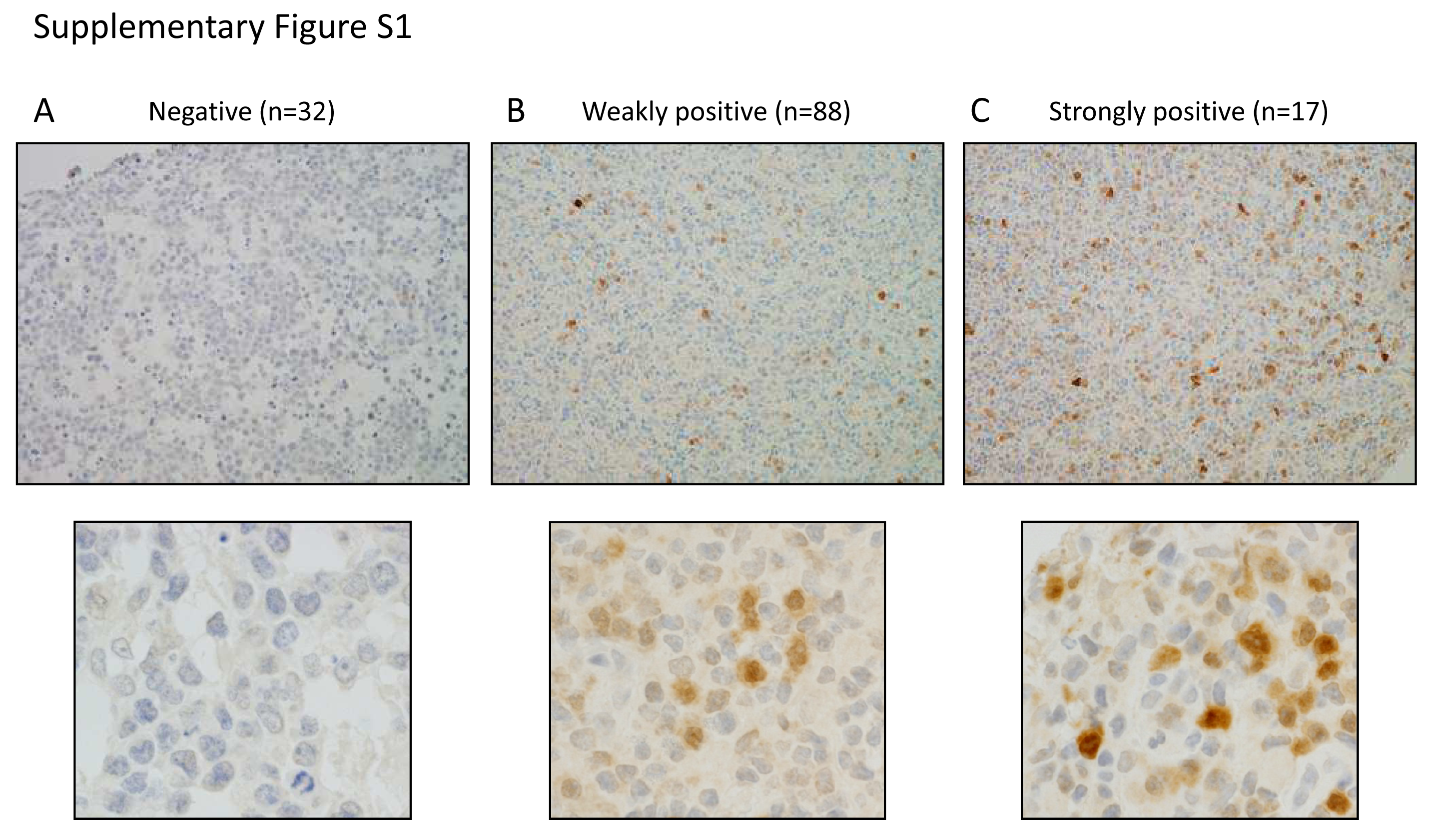

Supplement: Figure S1 — PIM2 protein in tumoral samples from PTCL patients. Representative immunohistochemical stainings for PIM2 (A) negative (<5% positive cells), (B) weakly positive (5–20% positive cells) and (C) strongly positive (>20% positive cells) samples from PTCL patients, specifically, a PTCL-NOS and two AITL, respectively (upper panels at 20X magnification and lower panels at 100X magnification). (TIF) [file pone.0112148.s001.tif]

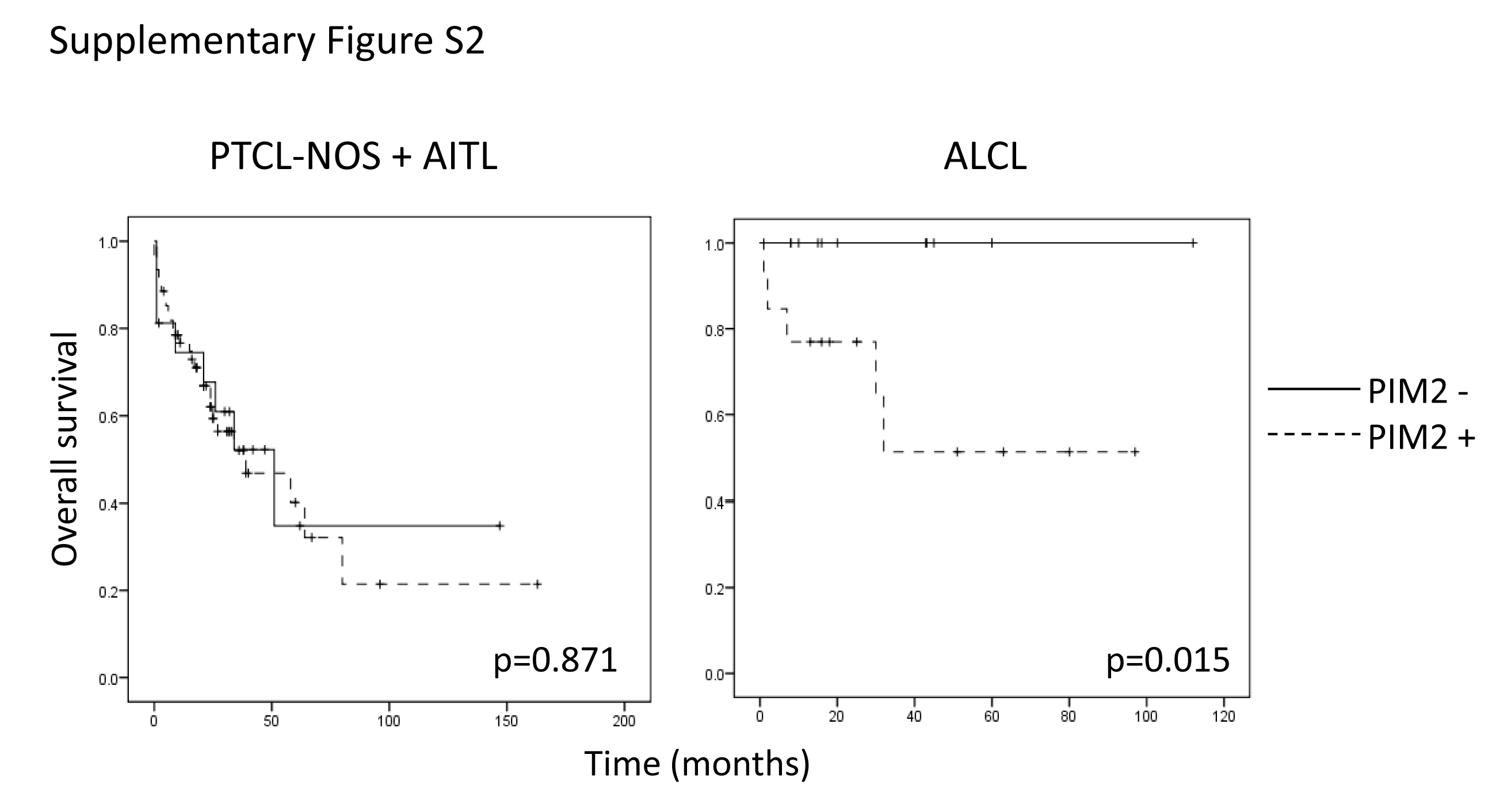

Supplement: Figure S2 — Association between PIM2 protein expression and overall survival in PTCL patients. PIM2 protein (both weak and strong signal) was significantly associated with worse overall survival in ALCL (n = 27), but not in the PTCL-NOS (n = 42) + AITL (n = 39) subgroups. (TIF) [file pone.0112148.s002.tif]

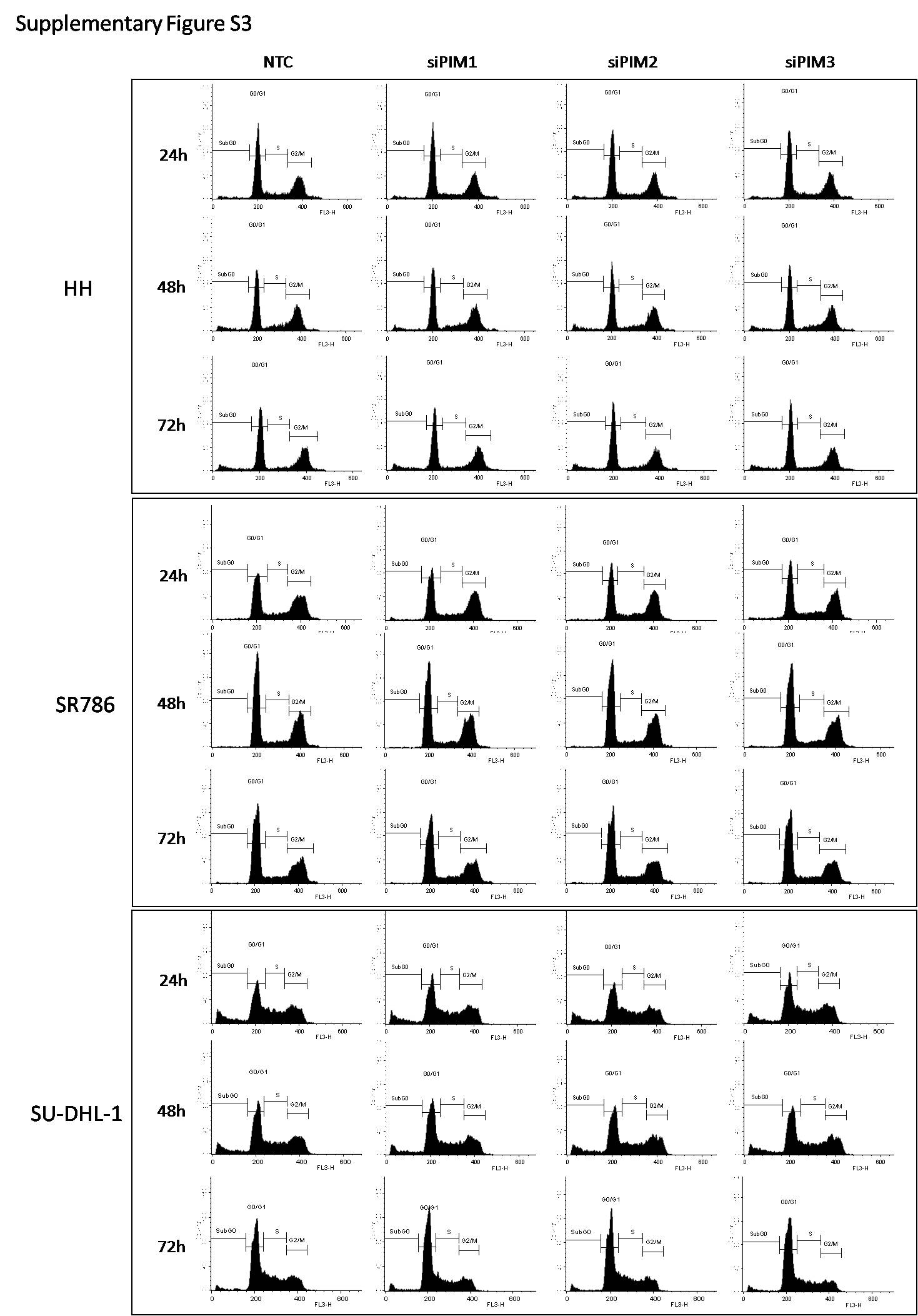

Supplement: Figure S3 — Effects of single PIM genetic knockdown on cell cycle in PTCL cell lines. Individual PIM gene inhibition (100 nM siRNA) did not induce cell cycle changes over the time. (NTC: non-template control). (TIF) [file pone.0112148.s003.tif]

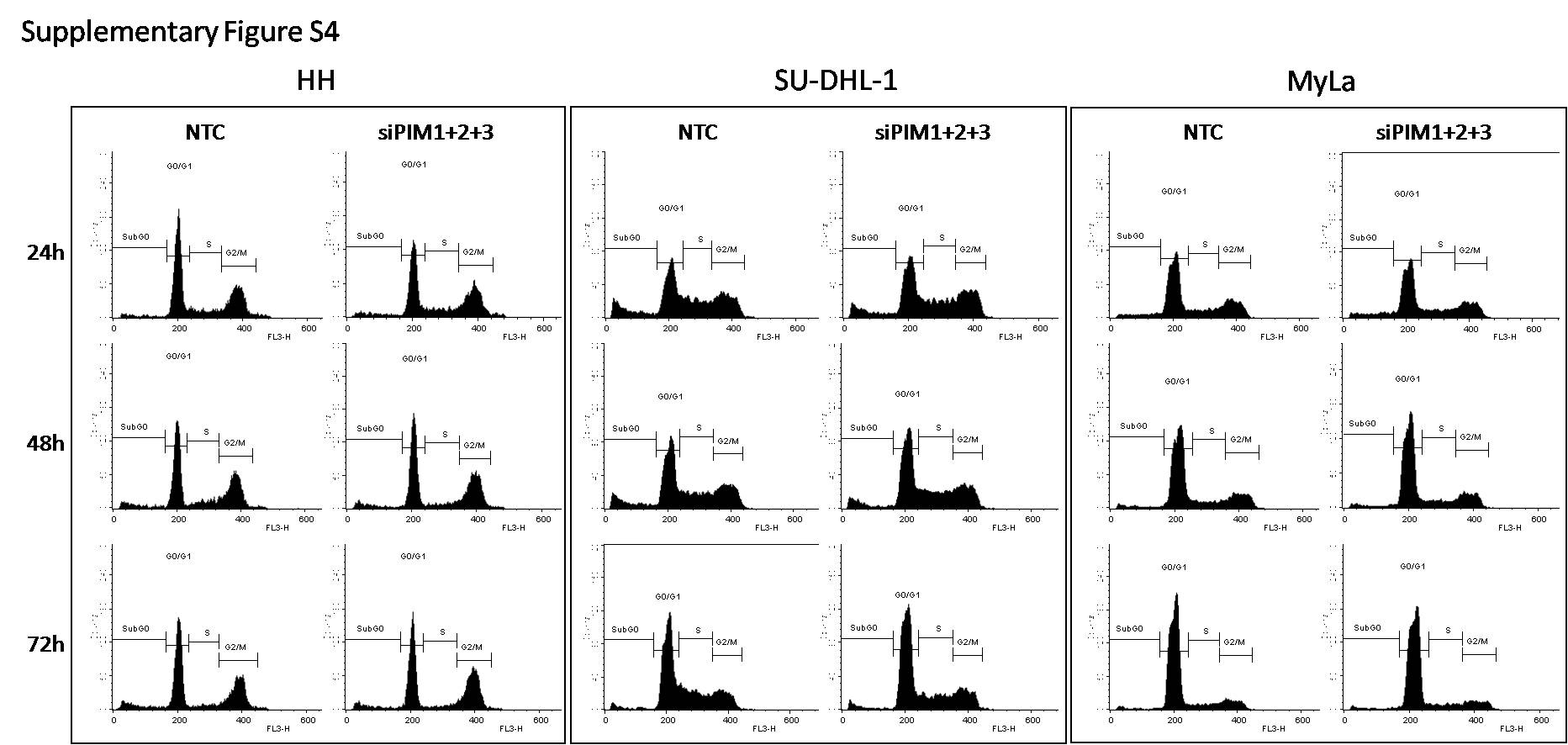

Supplement: Figure S4 — Effects of triple PIM genetic knockdown on cell cycle in PTCL cell lines. Simultaneous triple PIM1+PIM2+PIM3 gene inhibition did not induce cell cycle changes over the time. (NTC: non-template control). (TIF) [file pone.0112148.s004.tif]

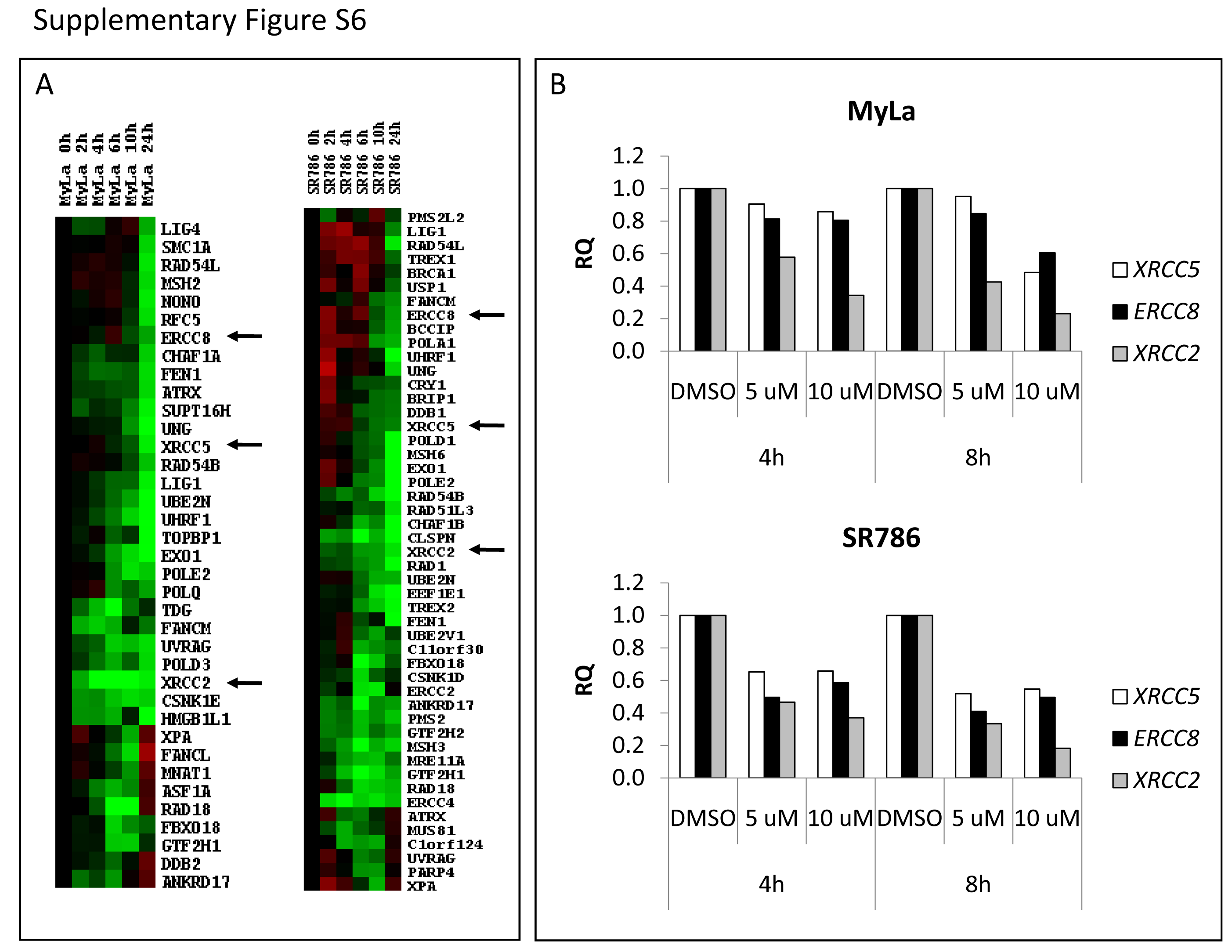

Supplement: Figure S6 — Downregulation of DNA damage repair signaling by the pharmacological pan-PIMi. (A) Heat-map showing an overall downregulation of genes involved in DNA damage repair machinery driven by the pharmacological pan-PIMi (10 µM at indicated times) in both MyLa and SR786 cell lines. These expression changes were significant (FDR<0.05), and extracted from Table S3. Some important genes, such as ERCC8, XRCC2 and XRCC5 (highlighted by arrows) were randomly selected to be validated. (B) Validation of microarray data by RT-qPCR. The expression of ERCC8, XRCC2 and XRCC5 genes was confirmed to be reduced in a time- and dose- dependent manner after pan-PIMi treatment in MyLa and SR786 cell lines. RQ, relative quantification, was calculated as described in the Methods section as RQ = 2−ΔCt. (TIF) [file pone.0112148.s006.tif]

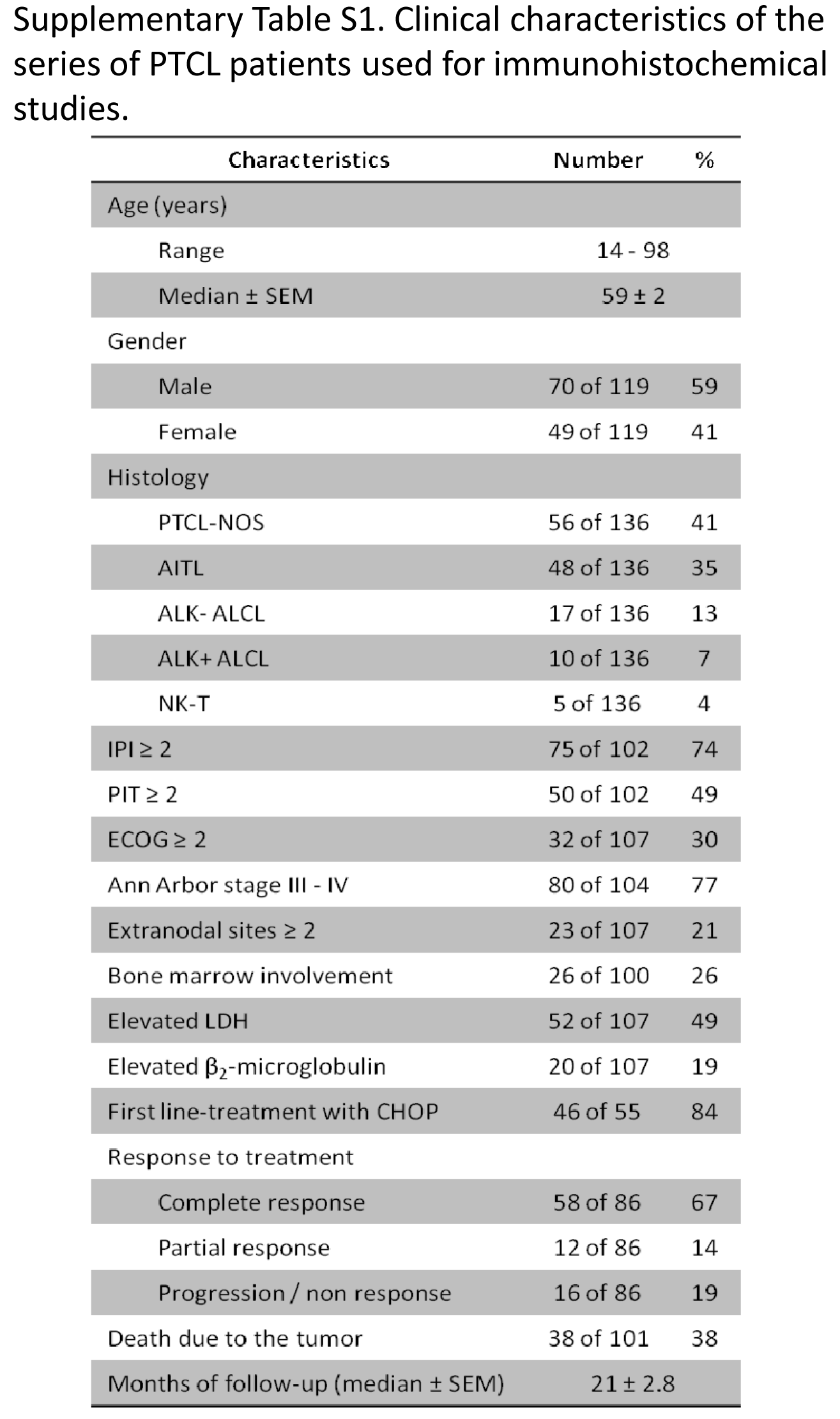

Supplement: Table S1 — Clinical characteristics of the series of PTCL patients used for immunohistochemical studies. PIM2 protein expression was explored in 136 PTCL patients. (PTCL-NOS: peripheral T cell lymphoma not otherwise specified; AITL: angioimmunoblastic T cell lymphoma; ALCL: anaplastic large cell lymphoma; NK-T: natural killer T cell lymphoma; IPI: international prognostic index; PIT: prognostic index for peripheral T-cell lymphoma, unspecified; ECOG: Eastern Cooperative Oncology Group; LDH: lactate dehydrogenase). (TIF) [file pone.0112148.s007.tif]

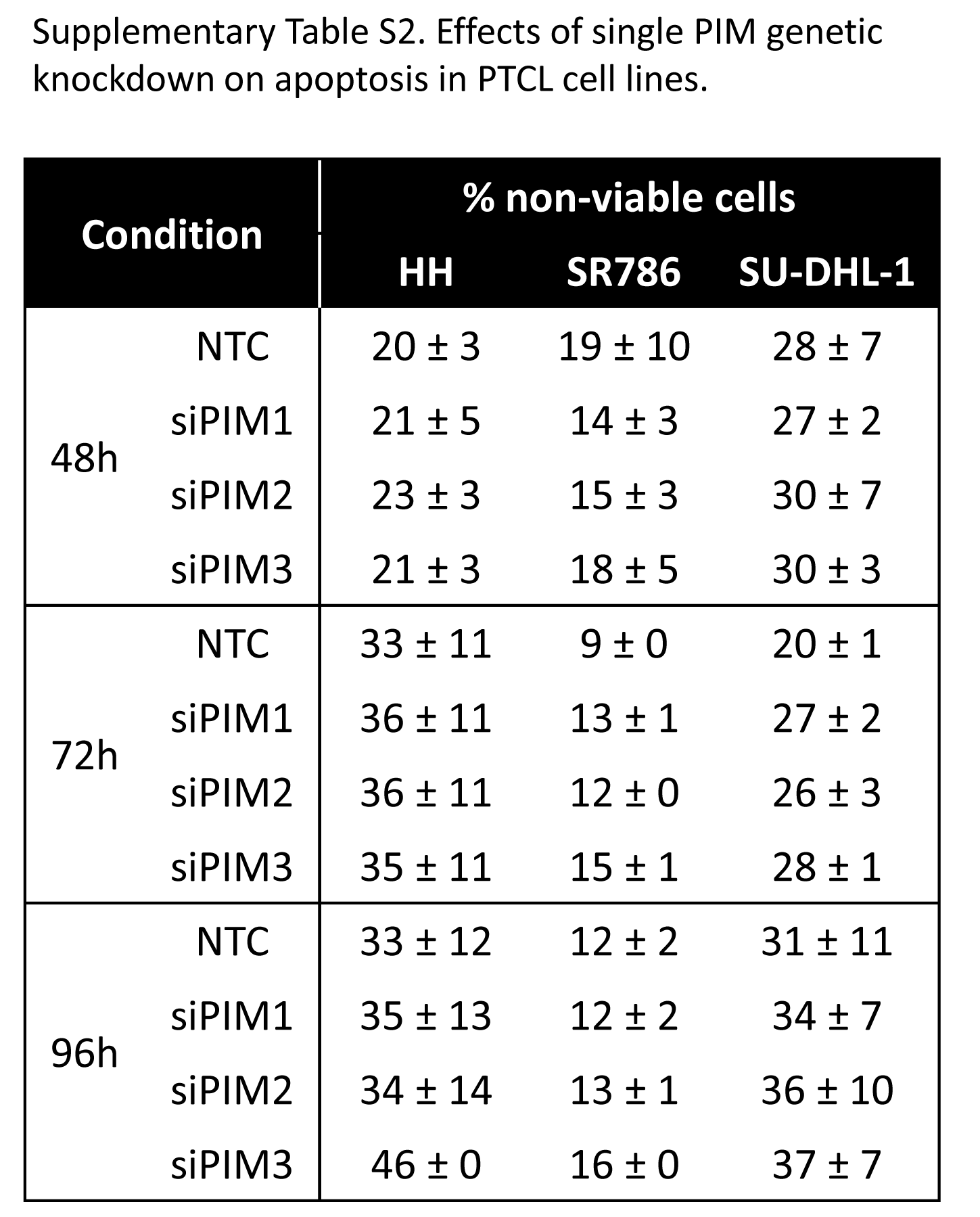

Supplement: Table S2 — Effects of single PIM genetic knockdown on apoptosis in PTCL cell lines. Individual PIM gene inhibition did not induce apoptosis over the time. The percentage of non-viable cells was calculated as Annexin V+/PI− plus Annexin V+/PI+ cells. (NTC: non-template control). (TIF) [file pone.0112148.s008.tif]

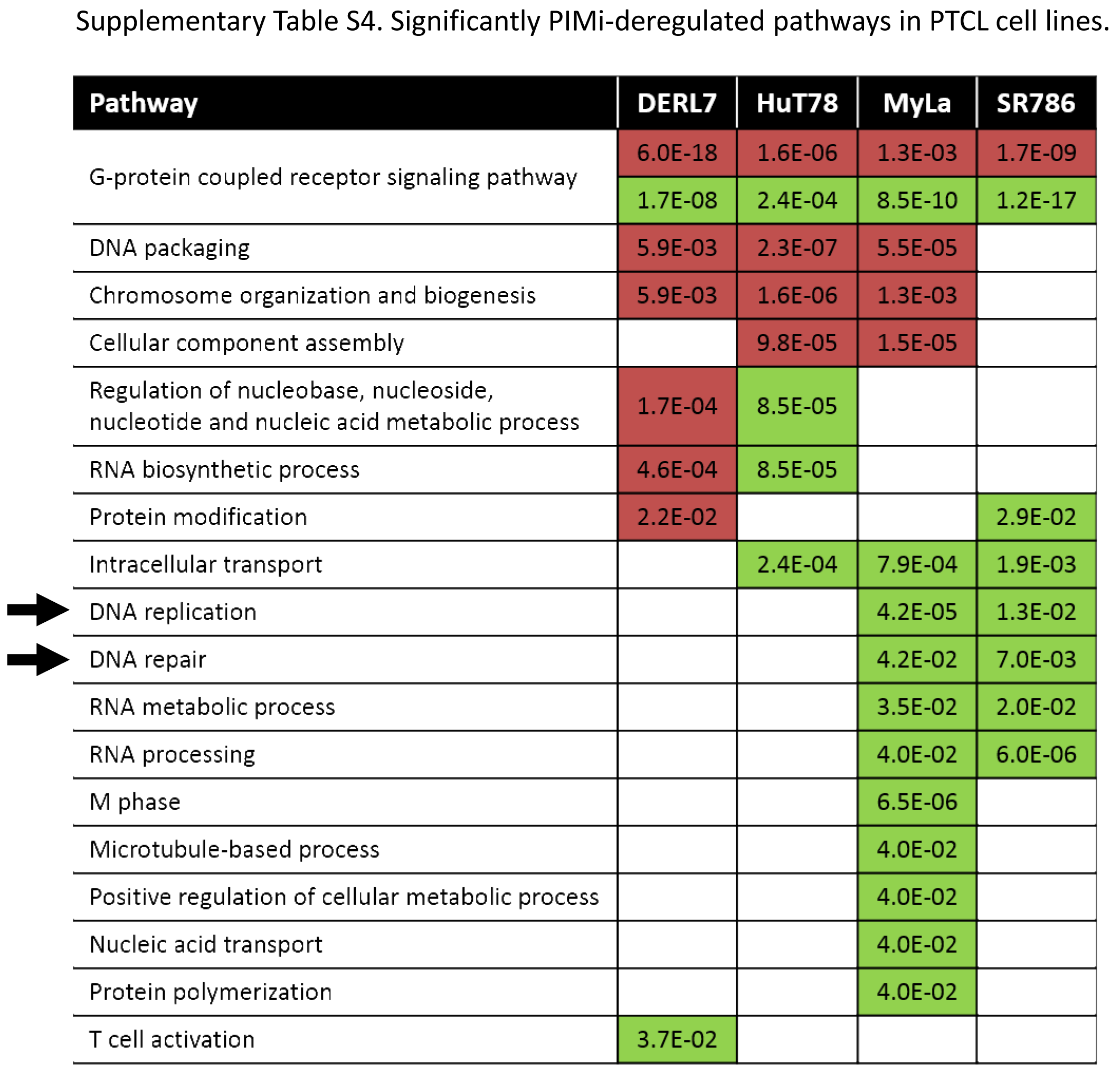

Supplement: Table S4 — Significantly PIMi-deregulated pathways in PTCL cell lines. Differentially expressed genes in each cell line upon pan-PIMi treatment identified by STEM (FDR<0.05) were applied to FatiGO to look for their functions. Significant biological processes at level 6 are shown (numbers indicate adjusted p-values). Red, green and white colors represent upregulation, downregulation and no significant deregulation, respectively. DNA-related processes are highlighted with arrows. (TIF) [file pone.0112148.s010.tif]
