# Supplementary material for: PIM Kinases as Potential Therapeutic Targets in a Subset of Peripheral T Cell Lymphoma Cases
Source: PLoS One. 2014 Nov 11;9(11):e112148. doi: 10.1371/journal.pone.0112148 (PMC4227704; doi:10.1371/journal.pone.0112148)

Supplementary Figure S5A

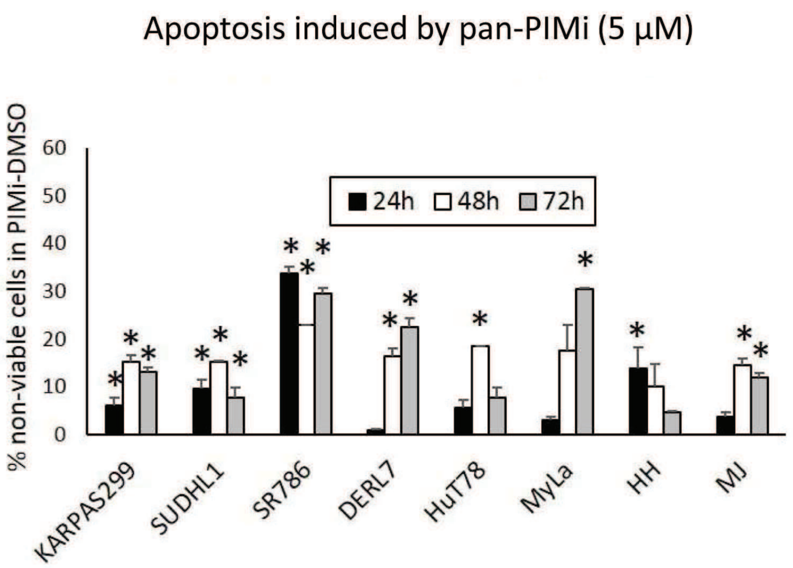

Supplementary Figure S5B

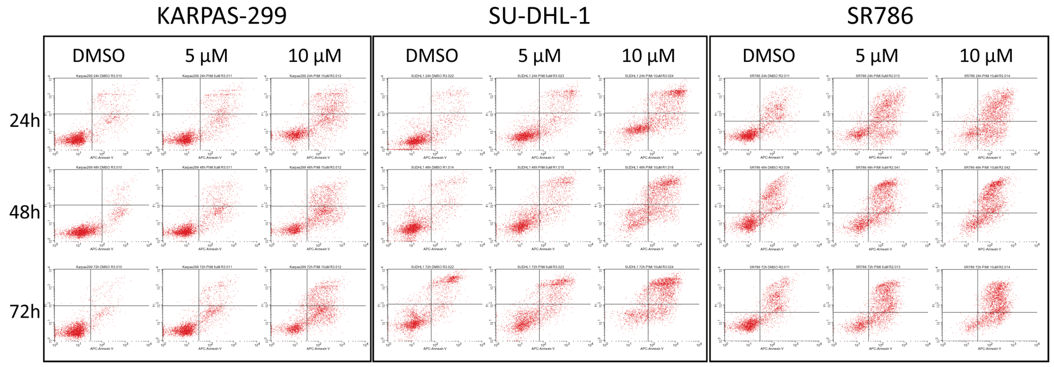

Supplementary Figure S5C

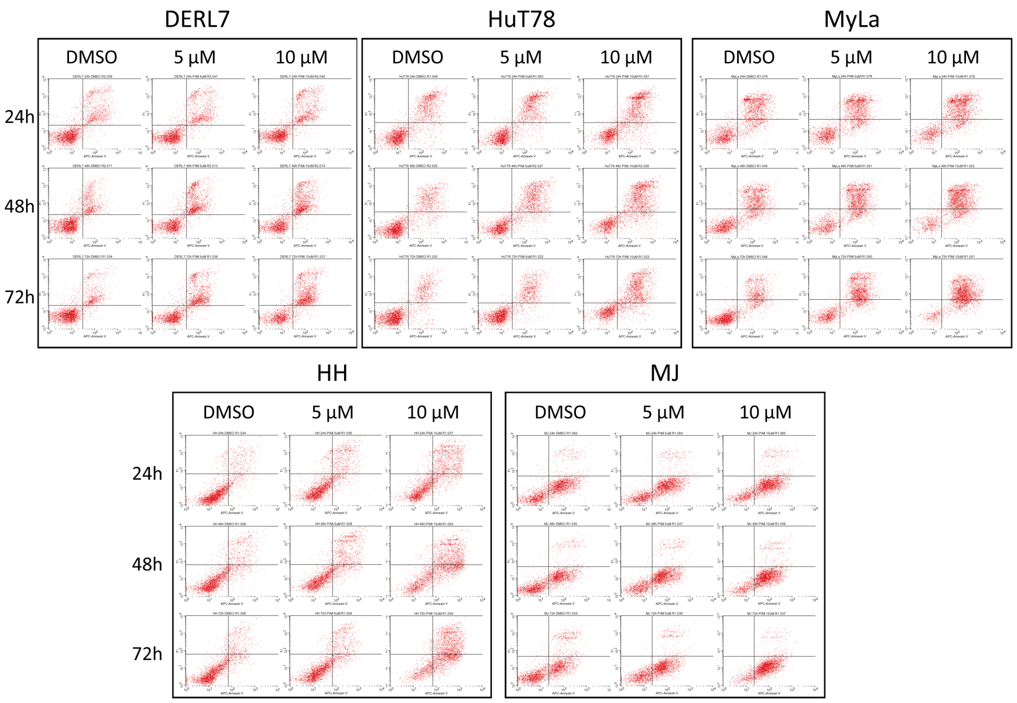

Supplementary Figure S5D

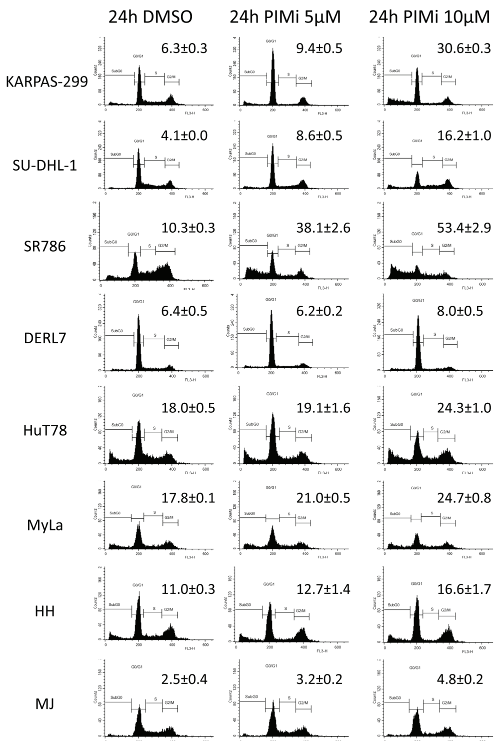

Supplement: Figure S5 — Effects of the pharmacological pan-PIMi on PTCL cell survival. (A) PTCL cell lines were treated with 5 µM of pan-PIMi for 24–72 h and effects on apoptosis were measured by flow cytometry. The percentage of non-viable cells was calculated as Annexin V+/PI− plus Annexin V+/PI+ cells in the PIMi-treated condition minus the DMSO-treated control. The pan-PIMi ETP-39010 strongly induced apoptosis in a time-dependent manner in all PTCL cell lines (*, p<0.05, from comparison with DMSO-treated cells). (B) Original scatter plots from FACS characterizing the effect of the pharmacological pan-PIMi on apoptosis in ALK+ ALCL cell lines: the X axis represents Annexin V staining and the Y axis represents PI staining. Representative plots from 3 independent experiments. (C) Original scatter plots from FACS characterizing the effect of the pharmacological pan-PIMi on apoptosis in other PTCL cell lines: the X axis represents Annexin V staining and the Y axis represents PI staining. Representative plots from 3 independent experiments. (D) The pan-PIMi (24 h) did not promote cell cycle arrest at any phase, but a direct increase in the subG0 fraction, as indicated numerically (mean ± SEM), especially in ALK+ ALCL cell lines (KARPAS-299, SU-DHL-1 and SR786). (PDF) [file pone.0112148.s005.pdf]
