# Supplementary material for: PIM Kinases as Potential Therapeutic Targets in a Subset of Peripheral T Cell Lymphoma Cases
Source: PLoS One. 2014 Nov 11;9(11):e112148. doi: 10.1371/journal.pone.0112148 (PMC4227704; doi:10.1371/journal.pone.0112148)
Supplement: Methods S1 — Additional detailed methodology. (DOC) [file pone.0112148.s011.doc]

**Supplementary Methods**

*Microarray hybridization*

RNA from each sample and Universal Human Reference RNA (Stratagene, La Jolla, CA) was reverse-transcribed using a Low RNA Input Fluorescent Linear Amplification kit (Agilent Technologies, Inc., Santa Clara, CA) for 2 h at 40 ºC. Equal amounts of Cy3- and Cy5-labeled amplified RNA were hybridized for 17 h at 65 ºC and 10 rpm. Finally, the slides were scanned using a G2565BA Microarray Scanner System (Agilent Technologies, Inc.).

*Bioinformatics analysis of the PTCL patient series*

The gene expression profiles in frozen tumoral samples from 38 PTCL patients and 6 reactive lymph nodes were compared. PTCL samples included: 20 PTCL-not otherwise specified (PTCL-NOS), 15 angioimmunoblastic T cell lymphomas (AITL) and 3 anaplastic large cell lymphomas (ALCL, 1 ALK+ ALCL and 2 ALK- ALCL). The research was approved by the Hospital Universitario Marqués de Valdecilla (Santander, Spain) ethics committee. Differentially expressed genes (False Discovery Rate, FDR, < 0.05) were identified by an independent-samples t-test (http://pomelo2.bioinfo.cnio.es/). Gene Set Enrichment Analysis (GSEA[1] http://www.broadinstitute.org/gsea/index.jsp) was used to explore the correlation between the expression of *PIM1* and *PIM2* and sets of genes grouped according to their cellular functions, as follows: GSEA ranked all significantly altered genes in PTCL *versus* reactive lymph nodes according to its correlation with *PIM1* or *PIM2* expression. Then, GSEA interrogated each gene belonging to every pathway in order to find whether it appeared positively or negatively correlated. Every time GSEA found a gene from a given pathway increased the Enrichment Score: the higher this value, the stronger the correlation. We used Biocarta (http://www.biocarta.com/), KEGG (http://www.genome.ad.jp/kegg/), Ingenuity Pathway Analysis (http://www.ingenuity.com/) and other sources publically available through the Molecular Signature Database (http://www.broad.mit.edu/gsea/msigdb/index.jsp) to generate the gene set database.

*Data analysis*

Data were obtained from scanned images using Feature Extraction v9.0 software (Agilent Technologies, Inc.). Briefly, data were normalized and preprocessed using the Gene Expression Pattern Analysis Suite (GEPAS v3.1, http://gepas3.bioinfo.cipf.es)[[2]](#_ENREF_4). Genes differentially expressed under each condition were identified using Short Time-series Expression Miner (STEM[[3]](#_ENREF_5), http://www.cs.cmu.edu/~jernst/stem/). An FDR < 0.05 was considered significant. These genes were hierarchically clustered using Cluster/Treeview[[4]](#_ENREF_6) (http://rana.lbl.gov/EisenSoftware.htm). Functional analysis was carried out by FatiGO (http://babelomics.bioinfo.cipf.es/functional.html) using Gene Ontology categories (biological processes at level 6).

*Immunofluorescence and immunohistochemistry*

After treatment with 10 μM pan-PIMi for 24 h, MyLa cells were passed onto a slide by a Cytospin (Thermo Scientific, Waltham, MA), fixed and permeabilized with acetone for 10 min. For immunofluorescence, slides were incubated with the γH2A.X antibody for 45 min, and after washing, with the secondary antibody (Alexa Fluor 555, Life Technologies) at 1:200 for 45 minutes. Slides were mounted using Vectashield mounting medium (Vector Laboratories Inc, Burlingame, CA) containing DAPI for nuclei visualization. Images were obtained under a fluorescence microscope (Axio Imager Z1, Zeiss, Oberkochen, Germany).

PIM2 protein expression was investigated by immunohistochemistry in a series of 136 formalin-fixed and paraffin-embedded PTCL patients. Briefly, an initial automated dewaxing and rehydration step followed by heat induction (100 °C for 20 min) were performed. Heat-induced antigen retrieval was performed using pH 8.8 ethylenediaminetetraacetic acid (EDTA)-based ready-to-use solution (Leica Microsystems, Wetzlar, Germany). Slides were subsequently incubated with 3% hydrogen peroxide (5 min), a 1:40 dilution of the anti-PIM2 primary antibody (#4730, Cell Signaling Technology Inc, Danvers, MA) for 20 min, a postprimary blocking reagent (to prevent nonspecific polymer binding) (8 min), horseradish peroxidase-labeled polymer (8 min), and diaminobenzidine substrate (10 min). All reagents were components of the Bond Polymer Refine detection system (Leica Microsystems, Wetzlar, Germany). PTCL samples were categorized as negative, weakly positive or strongly positive for PIM2 expression, when < 5 %, 5 – 20 % or > 20 % positive cells were present, respectively.

*Protein extraction, antibodies and western blot*

Cells untreated and treated as indicated were harvested and washed twice in phosphate-buffered saline for protein extraction. Briefly, cells were lysed using RIPA buffer (Sigma-Aldrich, St Louis, MO) containing protease and phosphatase inhibitors (Roche, Basel, Switzerland) for 30 min on ice and centrifuged. The protein fraction in the supernatant was then recovered and quantified using the Bio-Rad Protein Assay Dye Reagent Concentrate (Bio-Rad Laboratories Inc., Hercules, CA), in accordance with the manufacturer’s recommendations.

For western blot, 60 μg proteins were resolved by SDS-PAGE in a 12 % gel and transferred to a nitrocellulose membrane (Millipore, Billerica, MA). Membranes were blocked in 5 % bovine serum albumin and incubated with primary antibodies. They were then incubated with secondary antibodies (Alexa 680 nm or Alexa 800 nm, Rockland, Gilbertsville, PA) and scanned with an Odyssey Infrared System Scanner (LI-COR, Biosciences, Lincoln, NE). The primary antibodies for western blots used were obtained as follows: PIM1 (#3247), PIM2 (#4730), PIM3 (#4165), p-4E-BP1(Thr37/46) (#2855) and total 4E-BP1 (#9452) from Cell Signaling Technology Inc.; Caspase-3 (#40924) from Active Motif (Carlsbad, CA); BCL2 (#610538) from BD Biosciences (Franklin Lakes, NJ); γH2A.X(S139) (05-636) from Upstate-Millipore; ALK (IR641) from Dako (Glostrup, Denmark); and α-tubulin (T-6074) from Sigma-Aldrich.

**References**

1. Subramanian A, Tamayo P, Mootha VK, Mukherjee S, Ebert BL, et al. (2005) Gene set enrichment analysis: a knowledge-based approach for interpreting genome-wide expression profiles. Proc Natl Acad Sci U S A 102: 15545-15550.

2. Herrero J, Al-Shahrour F, Diaz-Uriarte R, Mateos A, Vaquerizas JM, et al. (2003) GEPAS: A web-based resource for microarray gene expression data analysis. Nucleic Acids Res 31: 3461-3467.

3. Ernst J, Bar-Joseph Z (2006) STEM: a tool for the analysis of short time series gene expression data. BMC Bioinformatics 7: 191.

4. Eisen MB, Spellman PT, Brown PO, Botstein D (1998) Cluster analysis and display of genome-wide expression patterns. Proc Natl Acad Sci U S A 95: 14863-14868.
